# Physicochemical Rationale of Matrix Effects Involved in the Response of Hydrogel-Embedded Luminescent Metal Biosensors

**DOI:** 10.3390/bios14110552

**Published:** 2024-11-13

**Authors:** Elise Rotureau, Christophe Pagnout, Jérôme F. L. Duval

**Affiliations:** 1 Université de Lorraine, CNRS, LIEC, F-54000 Nancy, France; jerome.duval@univ-lorraine.fr; 2 Université de Lorraine, CNRS, LIEC, F-57000 Metz, France; christophe.pagnout@univ-lorraine.fr

**Keywords:** whole-cell metal bioreporters, bioluminescence, hydrogel matrices, metallic contaminants, speciation, electrostatics, photoactivity, nutrients, oxygen resource

## Abstract

There is currently a critical need for understanding how the response and activity of whole-cell bacterial reporters positioned in a complex biological or environmental matrix are impacted by the physicochemical properties of their micro-environment. Accordingly, a comprehensive analysis of the bioluminescence response of Cd(II)-inducible PzntA-*luxCDABE Escherichia coli* biosensors embedded in silica-based hydrogels is reported to decipher how metal bioavailability, cell photoactivity and ensuing light bioproduction are impacted by the hydrogel environment and the associated matrix effects. The analysis includes the account of (i) Cd speciation and accumulation in the host hydrogels, in connection with their reactivity and electrostatic properties, and (ii) the reduced bioavailability of resources for the biosensors confined (deep) inside the hydrogels. The measurements of the bioluminescence response of the Cd(II) inducible-*lux* biosensors in both hydrogels and free-floating cell suspensions are completed by those of the constitutive *rrnB* P1-*luxCDABE E. coli* so as to probe cell metabolic activity in these two situations. The approach contributes to unraveling the connections between the electrostatic hydrogel charge, the nutrient/metal bioavailabilities and the resulting Cd-triggered bioluminescence output. Biosensors are hosted in hydrogels with thickness varying between 0 mm (the free-floating cell situation) and 1.6 mm, and are exposed to total Cd concentrations from 0 to 400 nM. The partitioning of bioavailable metals at the hydrogel/solution interface following intertwined metal speciation, diffusion and Boltzmann electrostatic accumulation is addressed by stripping chronopotentiometry. In turn, we detail how the bioluminescence maxima generated by the Cd-responsive cells under all tested Cd concentration and hydrogel thickness conditions collapse remarkably well on a *single plot* featuring the dependence of bioluminescence on free Cd concentration at the individual cell level. Overall, the construction of this *master curve* integrates the contributions of key and often overlooked processes that govern the bioavailability properties of metals in 3D matrices. Accordingly, the work opens perspectives for quantitative and mechanistic monitoring of metals by biosensors in environmental systems like biofilms or sediments.

## 1. Introduction

Whole-cell bioreporters are valuable tools and remarkable alternatives to conventional analytical techniques for detecting rapidly and selectively a given analyte in various environmental samples, and for addressing the bioavailability and possible toxicity of that analyte [1,2]. Bioreporter technology employs bacterial cells engineered with molecular recognition elements to sense analytes in the environment, like organic and inorganic contaminants. Genetic construction is achieved by the fusion of an analyte-responsive gene promoter to a gene coding for a protein that can be quantified easily. The measurable output—e.g., a chemical, optical or electrochemical signal—informs on the level of the targeted analyte felt by the microorganism once it has been internalized [3,4,5]. Thanks to a variety of well-characterized promoters used for genetic manipulation and construction purposes, whole-cell bioreporters have been designed to detect a broad spectrum of analytes in aqueous media, including mercury [6], cadmium [7,8], chromium [9], lead [10] or organics [11,12].

Despite the large use of bioreporter cells for the detection of metals in, for example, biomedical or environmental applications, the interpretation of the signal they produce over time and the conversion of that signal into a quantitative information on metal bioavailability remain topics of interest. Traditionally, bioreporter responses measured in samples of interests are converted into a ‘bioavailable metal fraction’ using a linear concentration–signal relationship obtained from calibration curves [13,14]. This strategy suffers, however, from a number of limitations that can lead to the erroneous evaluation of metal concentration. Amongst them, the use of referenced ‘standard’ solutions may poorly mimic the nutrient background of the samples to be analyzed whereas the supply of energy from the medium to the sensing cells is one of the main aspects that affects the metabolic functioning of whole-cell bioreporters and, in turn, their response in terms of signal amplitude and temporal variations [15,16,17]. A recent work by Delatour et al. [18] describes how sequential modes of bioluminescence emissions over time translate the cells’ ability to (re)allocate bioavailable energy resources (e.g., amino acids and sugars) in order to sustain light production. The resulting multimodal bioluminescence peak emission then reflects the strategies implemented by the cells to overcome the gradual depletion of some essential nutrients in the course of time and the ensuing operability of different metabolic pathways for optimal exploitation of the available nutrients. Other approaches commonly adopted to interpret signals by metal bioreporters are based on popular thermodynamic models such as the Biotic Ligand Model (BLM) or the Free-Ion Activity Model (FIAM) [19,20]. The BLM and FIAM are generally adopted to compute metal speciation in solution and connect empirically this speciation to metal bioaccumulation and biosensor maximal output over time. These models assume that the metal internalization step limits the kinetics of metal biouptake, which leads to the consideration that the metal species absorbed at the internalization sites of the membrane of the sensing cells are in equilibrium with the free metal species in bulk solution [21,22]. Accordingly, the applicability of the BLM and FIAM models is necessarily restricted to situations where the metal complexes in the medium are non-labile or inert, i.e., their dissociation and formation within the time window of their diffusion from the solution to the biological surface are very slow. As explained elsewhere [23], ignoring lability properties of metal complexes may lead to severe misinterpretation of biosensor signals, depending on cell type and physicochemical medium composition. Therefore, it is usually necessary to account for the dynamic reactivity (chemodynamics) of metal complexes in solution and the dynamics of metal partitioning at the biosensor/solution interface to achieve a mechanistic exploitation of the biosensor signal. This further includes the consideration of the possible depletion of metal ions from bulk solution during light emission, with dramatic implications of that process on the time response of the biosensors even under equilibrium conditions that legitimate a priori the use of the BLM [23].

The theoretical developments and supporting experimental data recently published in [15,16,17,18,23,24] help to decode the time-dependent signals produced by metal-sensing bioluminescent bacteria. Briefly, the modelling framework deciphers the way bacteria convert bioaccumulated metal fractions into light via the relevant cascade of extra- and intracellular events, with an account of the dynamics of metal biouptake at the microbial interface, the kinetics of the metal-mediated expression of the *lux*-reporter gene and the kinetics of luciferase production/degradation and photon emission. Models do further integrate the influence of the concentration and quality of nutrients in the medium on the (multimodal) bioluminescence emission patterns, recalling here that nutritional conditions impact necessarily the different processes leading to photon emission via an optimization of the cell energy budget. The interpretation of the metal-induced bioluminescence response in [18,24] was further supported by complementary experiments with constitutively luminescent reporter cells that sense metabolic activity. The response of the latter directly mirrors cell photoactivity over time, which reflects the sequence of operative metabolic pathways (e.g., the coupled stringent response and catabolite repression) adopted by the cells to exploit efficiently the available resources and produce light [24]. The latest theoretical formulation of the time-response of metal-detecting *lux*-bacterial sensors further details the connections between shape/amplitude of cell signal and the distribution of bioactive (i.e., internalized) metal species at the biosensor/aqueous medium interface following coupled reactive diffusion transport, bioaccumulation and depletion of metals from bulk solution. Interpreting biosensor response by means of this recent formalism enables the identification of scenarios where metal biouptake and cell response are limited by either metal internationalization or diffusion transport, and cases where (labile) metal complexes contribute to biouptake of free metals and, therewith, bioluminescence emission [23].

The above methodological and theoretical advances are restricted so far to luminescent bacterial metal sensors dispersed in aqueous media (planktonic cells) and to the corresponding analysis of metal content and bioavailability features. Their applicability to more complex and heterogeneous environmental matrices, like sediments or biogels [25,26] or synthetic hydrogels [27], remains challenging as it requires some fundamental knowledge on how the local nutritional and physicochemical conditions experienced by the cells inside these matrices affect their production of bioluminescence. This issue is very relevant as, in many natural habitats, microorganisms colonize surfaces in the form of biofilms that create favorable environmental conditions for their survival, with the cohesion of these biofilms ensured by secreted polymeric materials like exopolysaccharides. These macro- or microcolonies’ formation occurs in many natural systems such as soils, sediments and aquifers. The extracellular matrix is composed of a wide range of polymeric components or metabolites that can interact with essential and nonessential elements and which can consequently affect their bioavailability [28,29]. In addition to physical protection, the biofilm structure provides controlled diffusion conditions [30] which can limit the supply of nutrients to cells and lead to resource depletion. Bacteria are versatile organisms deploying several strategies to thrive in diverse environments and nutritional conditions thanks to their genetic plasticity, metabolic diversity and their capacity to establish various symbiotic relationships. To cope with resource scarcity, they may switch to different enzymatic systems or metabolic pathways to utilize less-preferred compounds. These microbial processes related to confined systems are also encountered in many other situations, e.g., sediment or soil pore water, and similar microbial behavior can be activated to withstand adverse conditions, including nutrient deficiencies.

In view of the above elements, this work reports the time-dependent bioluminescence response of Cd(II)-inducible *lux*-*E. coli.* reporters embedded in well-defined silica-based hydrogels over a large range of Cd concentration in solution and hydrogel thickness conditions. The objective is to provide a comprehensive physicochemical rationale of the matrix effects at the origin of the deviations of biosensors’ response measured in such hydrogels from the one obtained in free-floating cell configuration. A focus is given on the analysis of the hydrogel reactivity towards metal ions (i.e., metal speciation and electrostatic accumulation therein), the availability of nutrients in the hydrogels and their effects on the cells’ response over time. The hydrogels adopted in this work, generated by sol-gel synthesis, are known to maintain biocatalytic activity over days without loss of cell viability [31,32] and are well-suited substrates for array-based optical biosensing devices [32,33,34]. The hydrogel-biosensors’ assemblies studied were here exposed to a weak metal complexing medium containing cadmium ion, and light emission by the hydrogel-embedded biosensors was monitored for 17 h. *E. coli* sensors containing the cadmium-inducible PzntA promoter and the *luxCDABE* reporter gene were used for the experiments, together with another strain engineered to contain *luxCDABE*-reporter genes activated by the ribosomal RNA *rrnB* P1 promoter. This promoter is inhibited by (p)ppGpp alarmones produced by cells under stress conditions [15,24]. The corpus of experimental data collected for the biosensors hosted by the hydrogels was compared with that obtained in suspension (free-floating cells). In turn, this helped to investigate the paramount effects of biosensor immobilization in confined hydrogels systems on their bioluminescence response as a function of metal and nutrient bioavailability features. The approach detailed here to analyze the aforementioned matrix effects calls for the use of the electrochemical speciation technique and helps to unravel how hydrogel electrostatics, metal speciation and nutrient depletion mediate biosensor response in hydrogels. After correcting bioluminescence output for these matrix effects, we successfully generated a *master curve* that satisfactorily captures the luminescence response of an individual biosensor regardless of its physicochemical environment, from the free-floating cell suspension case to the hydrogel scenario. This result demonstrates that biosensors can be part of a robust quantitative methodology to probe metal bioavailability properties in 3D matrices with the account of the local physicochemical features of the close bacterial environment.

## 2. Materials and Methods

### 2.1. Design of Metal-Sensing Bacteria

An *Escherichia coli* strain JW3596 (Δ*rfaC*) was obtained from the Coli Genetic Stock Center, Yale University, USA [35]. Cadmium luminescent bioreporters, denoted here as JW3596-L, were engineered from the JW3596 strain by introducing the pZNT-*lux* plasmid that harbors a metal-inducible *zntA*-*luxCDABE* gene fusion [36]. A second luminescent whole cell biosensor, called JW3596-C, was used to monitor cell metabolic activity. This strain was obtained by introducing the plasmid pET28 *rrnB*P1 *luxCDABE* [37] in JW3596 cells, leading to the constitutive expression of luciferase under the transcriptional control of the ribosomal RNA *rrnB* P1 promoter.

### 2.2. Cell Growth Conditions

Lysogeny broth (LB) agar plates supplemented with selective antibiotics, i.e., kanamycin (30 µg mL^−^^1^), ampicillin (50 µg mL^−^^1^) for JW3596-L and kanamycin only (50 µg mL^−^^1^) for JW3596-C, were inoculated from a frozen glycerol cell stock stored at −80 °C. Single colonies of JW3596-L and JW3596-C were then collected from agar plates and aerobically cultured in liquid LB using a circular bath shaker (160 rpm, 37 °C) during 16 h. For both strains, cells were systematically washed twice by gentle centrifugation (5000 rpm) and dispersed in a poor metal-complexing glucose growth medium (nGGM). nGGM was composed of glucose (0.5%), MOPS (40 mM) MgCl_2_ (1 mM), NH_4_NO_3_ (12.5 mM), KNO_3_ (10 mM), K_2_SO_4_ (5 mM) and CaCl_2_.2H_2_O, β-glycerophosphate (5 mM), adjusted at pH 6.8 by addition of NaOH 1 M and sterilized by membrane filtering (0.22 µm polyethersulfone membrane from Millipore (Burlington, MA, USA)). Before sample preparation in microplates (in hydrogels or in suspensions), bacterial concentrations were initially fixed from optical density (OD_600nm_) measurements (at 600 nm) using a cell density meter (Ultrospec 10 from Amersham Biosciences (GE Healthcare, Amersham, UK). These concentrations were outside the optical density linear range. Therefore, to fix properly the cell concentration, measurement was performed in diluted solutions so as to bring the OD_600nm_ within the linear range of the cell density meter (from 0 to 1). For the samples of bacteria to be introduced in the hydrogels, the final optical density of the cell suspension on top of the hydrogel phase was fixed at a value of 13, i.e., by verifying that the OD_600nm_ of a 20-times-diluted solution was 0.65. For the bacteria samples kept in solution (planktonic cells), the final optical density of the cell suspension was fixed at 2, i.e., by verifying that the OD_600nm_ of a 2 times-diluted solution was equal to unity.

### 2.3. Hydrogel Preparation

**Figure 1:**
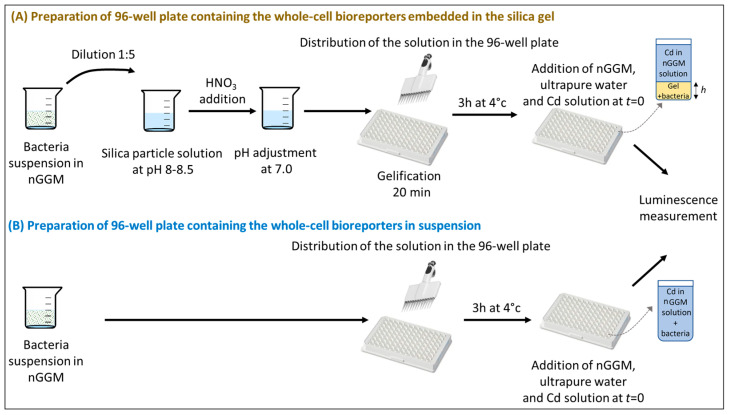
Procedures for the preparation of biosensor samples prior to bioluminescence measurement. In the situation where cells are embedded in silica hydrogels (**A**), a suspension of bacteria was prepared at a given cell concentration in nGGM medium, and it was then 5-times diluted in a solution of silica particles. The pH was adjusted to 7.0, which initiated the gelification process within 20 min. Prior to complete gelification, the so-obtained silica solution was added to the microplate. The hydrogel samples were left to stand for a period of three hours at 4 °C. Then, the addition of nGGM, ultrapure water and Cd solution was achieved prior to bioluminescence measurement (see text for details). Concerning the situation of biosensors in aqueous solution (**B**), a suspension of bacteria was prepared at fixed cell concentration in nGGM medium and left to stand for three hours at 4 °C. The addition of nGGM, ultrapure water and Cd solution was subsequently achieved prior to bioluminescence measurement. (See text for details).

The step-by-step preparation of the hydrogel samples with biosensors embedded therein is schemed in Figure 1. The reader is referred to the text below for details.

Briefly, a solution containing silica beads was initially prepared. This suspension contained 15.2% (*w*/*v*) Ludox^®^ HS40 (Ludox solution at 40% *w*/*w*, Fluka, Buchs, Switzerland), 8.1% (*w*/*v*) sodium silicate solution (Fluka), 5.8% (*w*/*v*) glycerol (99.5%, Sigma-Aldrich, Steinheim, Germany) and 0.24% (*w*/*v*) trehalose (from a 1% (*w*/*v*) solution prepared from trehalose powder (Sigma-Aldrich, ≥99%), in ultrapure MilliQ water (MilliporeSigma, Burlington, MA, USA). The solution pH was adjusted to 10.8 with the addition of sodium silica when necessary. Then, 4 mL of this solution was collected and the pH was initially adjusted to 8.0–8.5 by the addition of aliquots (20–50 µL) of HNO_3_ 1 M. A volume of 1 mL of the bacterial suspension was then introduced under stirring, and the pH was then adjusted to 7.2 ± 0.1. Lowering the pH led to the gelation of the silica beads solution after 20 min. Before reaching complete solidification, the solution was dispensed into a white and opaque 96-well microplate Cellstar^®^ from Greiner Bio-one (Frickenhausen, Germany). The volumes ranged from 26 µL to 62 µL with 8 µL incremental addition line by line. The microplate was stored at 4 °C for 3 h prior to the addition of Cd solution in nGGM 1:5 diluted and the start of the bioluminescence measurements.

### 2.4. Bioluminescence Assays

After 3 h of being stored, wells were filled with ultrapure MilliQ water and nGGM solution to obtain a final volume of 180 µL (Figure 1). The addition of 20 µL of Cd(NO_3_)_2_, ten times more concentrated than the final desired concentration, was then performed to obtain a total Cd concentration in each well ranging from 0 to 400 nM (concentration range relevant for the experiments on the biosensors embedded in the hydrogels). This value of the metal concentration refers to the total amount of metal divided by the whole sample volume in the well, which thus includes the hydrogel volume and the volume of the aqueous supernatant. Right after the addition of the Cd solution, the microplate was placed in a piece of luminometer equipment (SAFAS Xenius luminometer, SAFAS, Monaco city, Monaco). Bioluminescence measurements (in photon counting mode) were achieved every 10 min at 22 °C over 17 h. An orbital shaking was applied before each measurement (10 s, 3 mm amplitude and 10 Hz frequency).

We confirmed the applicability of the Beer–Lambert law by verifying the linearity between the intensity of light emitted by the hydrogel-embedded biosensors and the hydrogel thickness at constant cell density. To do so, 20 min after their preparation, we measured the light from hydrogels of varied thickness containing constitutive luminescent bacteria in contact with a 1:5 nGGM medium in the absence of Cd(II). The range of linearity was confirmed for values of the hydrogel thickness between 600 µm and 1800 µm. In addition, we confirmed the linearity between the transmitted light and the hydrogel thickness, which warranted homogeneous diffusion of the produced photons all across the hydrogel along their way to the collector. To do so, *E. coli* bacteria expressing Green Fluorescent Protein (GFP) were used and embedded in the silica hydrogels following the same protocol as the one described above (Figure 1). The hydrogel sample was placed in black 96-well microplates Cellstar^®^ from Greiner Bio-one with an optically clear flat well bottom. An excitation source illuminated the sample at the bottom, and the emitted fluorescence was collected at the top by a photomultiplicator (PM). The sample was excited at 470 nm and the emission spectrum was recorded for every gel thickness tested in this work. The percentage of emitted light was taken at the maximal emission wavelength (i.e., 512 nm), with a PM set at the same value for each measurement. According to the linear relationship obtained between the % of fluorescence emission and the gel thickness, we concluded that no multiple-photon diffusion took place and that photon diffusion was similar regardless of the location of the emission source in the hydrogel, in agreement with the linear relationship established on the basis of the bioluminescence measurements.

For the bioluminescence assays performed on planktonic biosensors in an aqueous medium (i.e., in the absence of hydrogels), bacteria solution with OD_600nm_ adjusted to 2 was distributed in the microplate wells at various volumes in order to obtain a final optical density ranging from 0.18 to 0.58 after dilution. After a rest time of 3 h at 4 °C, the wells were filled with ultrapure MilliQ water and nGGM solution to obtain a final volume of 180 µL. Then, 20 µL of Cd(NO_3_)_2_ ten times more concentrated than the final desired concentration was added to obtain a total Cd concentration in each well ranging from 0 to 40 nM. (This concentration range is relevant for the experiments that did not involve the hydrogels.) Then, bioluminescence measurements were performed following the protocol detailed above for the hydrogel-embedded biosensors.

To ensure the robustness and reproducibility of our findings, we systematically measured three independent replicates for each reported condition tested in this work. The data presented in this paper originate from a single representative experiment, and we stress that the observed variations of the maximal cell bioluminescence emitted over time did not exceed ca. 20% under the given Cd and cell concentration conditions: we attribute this deviation to inherent biological variability from one sample to another. We systematically observed that all replicates evidenced the same trends in response to parameter variations (Cd concentration, cell concentration, hydrogel thickness), which indicates that the reported data solidly capture the basic properties of the system. Regarding bioluminescence experiments in hydrogels conducted under given experimental conditions, we performed complementary measurements in solution using the same microplate as a control. An example of 3 experimental replicates measured for gel-embedded biosensors are given in Figure S1 of the Supporting Material (SM-A).

### 2.5. Analysis of Cd Partitioning Between Hydrogel and External Aqueous Solution

The analysis of Cd partitioning at the interface between the hydrogels and the external solution was performed using an electroanalytical technique called stripping chronopotentiometry, which we detail below together with the electrochemical setup we employed.

#### 2.5.1. Experimental Electrochemical Setup

The electrochemical apparatus includes an Ecochemie Autolab type III potentiostat controlled by GPES 4.9 software (Ecochemie, Utrecht, The Netherlands) connected to a voltammetry stand (Metrohm model 663VA, Metrohm, Herisau, Switzerland). The setup comprises three electrodes: a rotating glassy carbon disk (2 mm radius) coated with a thin mercury film electrode (TMFE working electrode), a Dri-ref-5 reference electrode from World Precision Instruments (Sarasota, FL, USA) and a counter glassy carbon electrode. The preparation of TMFE was repeated daily for each set of experiments according to the procedure detailed in part B of the Supporting Material (SM-B). The experiments described hereafter in Section 2.5.3 were repeated twice for two values of the hydrogel volume fraction, φ=0.13 and 0.26, where φ is defined by $\varphi =V^{gel}/\left(V^{gel}+V^{sol}\right)$ with $V^{gel}$ and $V^{sol}$ the volume of the hydrogel and solution, respectively.

#### 2.5.2. Cadmium Content Quantification by Stripping Chronopotentiometry

The determination of the metal content in solution was performed using stripping chronopotentiometry (SCP), a two-step electrochemical technique. The first step consisted of the deposition of metal in the TMFE via the application of a potential, Ed (−0.750 V vs. Ag/AgCl) for a fixed delay called *t*_d_ (45 s). Then, a constant oxidizing current, *I*_S_ (=0.239 A m^−2^) was applied until the potential reached a value well beyond the reoxidation transition plateau (0.40 V vs. Ag/AgCl for Cd). The analytical signal representing the time required for metal reoxidation was measured and led, in turn, to the evaluation of the total metal concentration in solution. The reader is referred to [38,39,40] for a detailed description of the functioning principles of SCP.

#### 2.5.3. Kinetics of Cd Partitioning at Hydrogel/Solution Interface

Prior to Cd quantification in solution and in the hydrogels, a SCP calibration was performed in a nGGM 1:5 medium to relate the total metal concentration to the SCP signal. Cd speciation in the nGGM medium of interest here was estimated with help of Visual Minteq-based computations [41], and a complementary experiment was performed to evaluate the concentration of free (not complexed) metal ions. Details can be found in the Supporting Material, part C. All in all, we determined that 70% of Cd existed in free form in nGGM 1:5 solution. It is emphasized that SCP measured the free metal fraction *plus* the labile Cd-complexes, i.e., those complexes whose dissociation and formation are infinitely fast as compared to the timescale for their diffusion from bulk solution to the electrode surface. In the case of nGGM media, we checked that approximately 95% of the total metal content was measured due to the high lability of the metal complexes formed with the anions of the background electrolyte adopted in the experiments. Accordingly, we performed SCP calibration directly in the nGGM 1:5 medium buffered at pH 7.2. Volumes of 2.6 or 5.2 mL of silica hydrogels, freshly prepared according to the procedure detailed in Section 2.3, were introduced in a disposable polystyrene beaker and supplemented with a nGGM 1:5 solution to obtain a total volume of 20 mL. Then, 150 µL of 10^−5^ M Cd(NO_3_)_2_ were added, and we subsequently monitored by electrochemistry the decrease of metal concentration in bulk solution over time as a result of the ongoing Cd accumulation in the hydrogels. Accordingly, the time dependence of the metal concentration in solution and in the hydrogel compartments could be determined using a calibration plot and applying the mass balance equation for the metal species.

## 3. General Theoretical Framework for the Interpretation of the Time-Dependent Bioluminescence by Whole-Cell Bioreporters

### 3.1. Nomenclature

**Figure 2:**
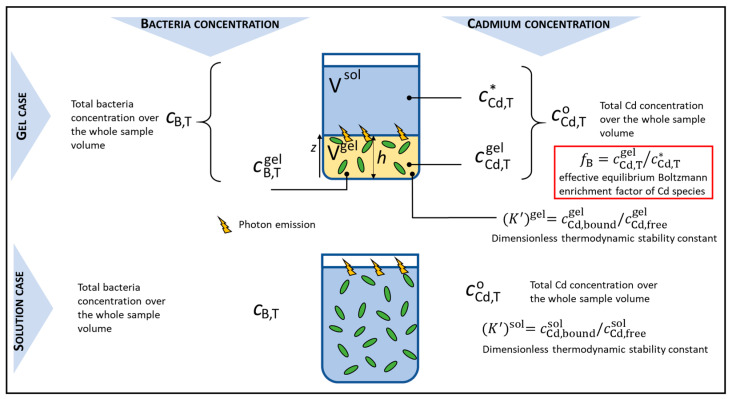
Definition of the nomenclature adopted to designate the total concentration of bacteria, the concentration of photoactive bacteria, the total concentration of Cd species and the concentration of free Cd(II) ions for the two experimental configurations of interest, i.e., bioluminescence measurements performed either in aqueous solution in the presence of a hydrogel phase hosting the biosensors (**top panel**) or in aqueous solution where biosensors are dispersed in the absence of hydrogel (**bottom panel**).

In this section, we introduce and define the nomenclature of the key biophysiochemical parameters that we shall refer to in our discussion of the time response measured for the biosensors dispersed in solution or embedded in hydrogel with varied thickness. For the sake of clarity, we report in Figure 2 a synopsis of these parameters together with their meaning. A glossary of the symbols adopted in this work is reported at the end of the paper.

The total concentration of bacteria (expressed in the number of cells per unit volume) applicable to the whole sample volume is here denoted as $c_{B,T}$, whether the sample refers to the solution or to the solution + hydrogel (Figure 2). The total cell concentration within the gel volume is further denoted as $c_{B,T}^{gel}$ and it is kept constant in this work. Therefore, for the situation where biosensors are embedded in the hydrogels (Figure 2, top panel), we have the relationship $c_{B,T}=c_{B,T}^{gel}\times \varphi$ where $\varphi =V^{gel}/\left(V^{gel}+V^{sol}\right)$ (cf. Section 2.5). The total cell concentration is evaluated from the optical density measured at 600 nm using the equivalence of 7.8 × 10^8^ cell mL^−1^ for OD_600nm_ = 1 [42]. For all bioluminescence assays reported in this work, the total cell concentration ($c_{B,T}$ and $c_{B,T}^{gel}$) is independent of time, i.e., there is no significant cell growth. We further introduce the concentration of photoactive cells [16,23,24] in the gel, denoted as $c_{B,p}^{gel}$, and we denote as $c_{B,p}$ the concentration of photoactive biosensors when dispersed in solution. As detailed in the theoretical section below, $c_{B,p}^{gel}$ and $c_{B,p}$ both depend on time *t* [16,23,24].

Cd concentration is denoted hereafter as $c_{\mathrm{Cd}}$ with the following specifications that are relevant especially for the discussion of the bioluminescence measurements involving the hydrogels. We introduce $c_{Cd,T}^{O}$, the total concentration of Cd over the whole sample volume, whether that volume refers to that of the aqueous solution in the absence of hydrogels, or to that of the aqueous solution + the hydrogel (Figure 2). Obviously, $c_{Cd,T}^{O}$ corresponds to the Cd concentration initially introduced in the sample. For the measurement situation where biosensors are embedded in the hydrogels, a partitioning of Cd will occur between the solution and the hydrogel phase. Accordingly, we differentiate $c_{\mathrm{Cd},T}^{*}$ and $c_{\mathrm{Cd},T}^{\mathrm{gel}}$, the total equilibrium concentrations of Cd in the solution positioned on top of the hydrogel and in the hydrogel, respectively.

In the silica hydrogels, we expect that Cd will distribute at equilibrium between free ionic species and other complexes formed with the negatively charged silica nanobeads precursors. In the absence of hydrogels (the planktonic case), nGGM 1:5 is a low metal-complexing medium so that free Cd ions will dominate. However, complexed Cd species are still present in a significant amount, as detailed in SM-C and below. To describe Cd metal complexation in both measurement configurations (presence and absence of hydrogels), we can formulate the dimensionless thermodynamic stability constant ${\left(K^{\prime }\right)}^{x}$ of the Cd complexes in the solution and in the hydrogel (x = sol and x = gel, respectively) according to

(1) $${\left(K^{\prime }\right)}^{x}=c_{\mathrm{Cd},\;\mathrm{bound}}^{x}/c_{\mathrm{Cd},\;\mathrm{free}}^{x}$$

where $c_{\mathrm{Cd},\;\mathrm{bound}}^{x}$ is the concentration of bound Cd species and $c_{\mathrm{Cd},\;\mathrm{free}}^{x}$ is the concentration of free (unbound) Cd ions. Equation (1) assumes that there is an excess of metal binding sites as compared to the total amount of metal, which is legitimate due to the high surface area developed by the silica nanobeads composing the hydrogels. In turn, the concentration of reactive sites at the silica nanobeads’ surface remains constant as it is not impacted by metal complexation.

### 3.2. Theoretical Formulation of the Bioluminescence Emission over Time: Case of Planktonic Biosensors

The bioluminescence produced over time (*t*) by metal-detecting whole-cell bacterial sensors dispersed in a aqueous solution of volume $V_{T}$, denoted as *Lum*(*t*) (in counts s^−1^) is related to the concentration of photoactive cells $c_{B,p}\left(t\right)$ (that differs from the total cell concentration, cf. [16,23,24]) via a convolution product which includes the terms associated with the dynamics of bioavailable metal transfer from the solution to the biosensor surface and to the kinetics of photon emission after metal internalization, i.e., [16,23,24]

(2) $$\mathrm{Lum}(t)=\psi \theta c_{B,T}\left(1-\gamma {\;S}_{a}c_{B,T}\right)J_{u}\left\{F\;\left(t\right)⊗{\bar{c}}_{B,p}\left(t\right)\right\}$$

where the total number concentration of bacteria $c_{B,T}$ (m^−3^) is defined in Section 3.1 (cf. Figure 2). Equation (2) is valid in the absence of metal depletion from the bulk solution during bioaccumulation and light production [23], and under conditions where there is insignificant cell growth over time. We verified that these conditions are met in this work. The symbol ⊗ in Equation (1) is the convolution product in the time domain, and the scalar ψ (in counts s^−1^ mol^−1^ m^5^) is defined by

(3) $$\psi =S_{a}V_{T}k_{\nu }k_{f}\tau_{q}K_{\mathrm{Hi}}^{-1}$$

where $S_{a}$ (m^2^) is the surface area of an individual sensing cell, $k_{\nu }$ (counts s^−1^ mol^−1^) is the kinetic constant for photon emission per mole of luciferase, $k_{f}$ (mol m^−3^ s^−1^) is the kinetic constant for luciferase production, $\tau_{q}$ (s) is the characteristic timescale over which bioluminescence is emitted by a given luciferase–luciferin complex, and $K_{Hi}$ (mol m^−3^) is the Hill constant linked to the affinity of the promoter (that controls the *lux*-reporter gene transcription) for the complex formed between intracellular Cd and the regulatory protein ZntR. The quantity ${\bar{c}}_{B,p}\left(t\right)$ (dimensionless) in Equation (1) is the number concentration of photoactive cells at time *t*, denoted as $c_{B,p}\left(t\right)$ (m^−3^), normalized by the maximum number concentration of photoactive cells $c_{B,p}^{\max}$ (m^−3^) the medium can sustain depending on the nutrients available therein [16,23,24]. As a first order approximation, we write $\theta =c_{B,p}^{\max}/c_{B,T}$ the ratio between maximum concentration of photoactive cells and total cell concentration (the latter includes both photoactive and photoinactive bacteria) [23]. As extensively detailed in [15,23], the term $\left(1-\gamma {\;S}_{a}c_{B,T}\right)$ accounts for the reduction of metal bioavailability due to the fraction of metal ions that is passively adsorbed at the cell surface (and thus not internalized), with γ (m) that refers to the affinity of metals for the passive adsorption sites at the cell membrane.

The function *F*(*t*) (dimensionless) involved in Equation (1) is detailed in [16,23], and it models the dynamics of metal partitioning at the solution/cell interface and the subsequent kinetics of the intracellular processes that lead to light emission. The reader is referred to [16,23] for extensive details on that function. Finally, the quantity $J_{u}$ stands for the metal biouptake flux (expressed in mol m^−2^ s^−1^). Within the framework of the Michaelis–Menten uptake mechanism, there is a fast Langmuirian adsorption of the internalizable Cd(II) ion species (the bioactive Cd form here) at the cell membrane transporter sites, followed by a rate-limiting metal internalization step with the kinetic constant $k_{int}$ (s^−1^). In metal biosensing studies with bacterial reporters, the linear Henry regime for metal bioaccumulation is applicable as it corresponds to a low coverage of the internalization sites by Cd and to an ensuing linearity between bioluminescence measured at *t* and the bioavailable metal concentration [16,23,24]. Under such conditions, $J_{u}$ is written [23]

(4) $$J_{u}=K_{H}k_{int}c_{\mathrm{Cd},\;\mathrm{free}}^{\mathrm{sol}}/\left(1+{\left(pBn\right)}^{-1}\right)$$

where $K_{H}$ (m) is the Henry coefficient for the adsorption of Cd ions on the transporter sites located at the cell membrane, and $c_{\mathrm{Cd},\;\mathrm{free}}^{\mathrm{sol}}$ (mol m^−3^) is the bulk concentration of free Cd ions (Figure 2, top panel). *p* (dimensionless) is a quantity that integrates the contribution of the metal complexes to the biouptake flux $J_{u}$ of free metal, and *p* depends on the lability of these complexes. The reader is referred to [23] for detailed expression of *p*. The dimensionless parameter Bn is the so-called Bosma number which compares the Cd diffusion conductance and the Cd internalization conductance defined by $a^{-1}D_{Cd}$ (m s^−1^) and $K_{H}k_{int}$ (m s^−1^), respectively, according to $Bn=a^{-1}D_{Cd}/\left(K_{H}k_{int}\right)$, with a the cell radius and $D_{Cd}$ the diffusion coefficient of Cd(II) in solution [16,23,24]. Equation (4) holds under stationary diffuse transport conditions of Cd(II) from the solution to the cell surface, and these conditions are rapidly achieved, i.e., within the timescale $a^{2}/D_{Cd}$ which is of the order of 1 ms for a ~ 1 μm and $D_{Cd}\;\sim {\;10}^{-9}$ m^2^ s^−1^ [43]. The extremes ${Bn}^{-1}\ll 1$ and ${Bn}^{-1}\gg 1$ correspond to the situations where metal bioaccumulation is kinetically limited by the metal internalization step and by the metal diffusion transfer from the bulk medium to the cell surface, respectively. Previous work [44] established that the biouptake of Cd(II) by *E. coli* Keio knock-out mutants (the strain from which the biosensors considered in this work are constructed) is rate-limited by the metal internationalization step and not by Cd diffusion from solution to the cell surface. Accordingly, we can write ${Bn}^{-1}\to 0$ in Equation (4), with the consequence that Cd complexes do not contribute to the metal biouptake flux $J_{u}$. Combining Equations (1)–(4), we then obtain after rearrangements

(5) $$\mathrm{Lum}(t)=\psi \theta \frac{K_{H}k_{int}}{1+{\left(K^{\prime }\right)}^{sol}}c_{B,T}\left(1-\gamma {\;S}_{a}c_{B,T}\right)c_{\mathrm{Cd},\;T}^{\mathrm{sol}}\left\{F\;\left(t\right)⊗{\bar{c}}_{B,p}\left(t\right)\right\}$$

where we used Equation (1) and the mass balance equation $c_{Cd,T}^{O}=c_{\mathrm{Cd},\;\mathrm{free}}^{\mathrm{sol}}+c_{\mathrm{Cd},\;\mathrm{bound}}^{x}$. Equations (2)–(4) refer to the situation of planktonic luminescent biosensors (bottom panel, Figure 2). Below, we detail how Equation (5) can be modified to tackle the more complex situation of hydrogel-embedded biosensors (Figure 2, top panel). To do so, we first need to discuss how hydrogel electrostatics impact Cd accumulation therein.

### 3.3. Determination of Boltzmann Cd Accumulation in the Hydrogel Due to Electrostatics

As a result of the negative charges carried by the hydrogel matrix prepared from silica nanobeads precursors, a Donnan potential is established between the hydrogel and the surrounding aqueous solution [45,46]. This potential can significantly govern the equilibrium distribution of ions inside and outside the hydrogel depending on the electrolyte concentration and the screening of the hydrogel charge by the background electrolyte ions. In particular, Donnan partitioning leads to the enrichment of cations in the hydrogel phase as compared to the outer solution. Accordingly, we define hereafter an *effective* equilibrium Boltzmann enrichment factor of Cd species, denoted as $f_{B}$, and defined by the ratio between the total Cd concentration in the hydrogel, $c_{Cd,T}^{gel}$, and the total Cd concentration in the bulk solution outside the hydrogel, $c_{Cd,T}^{*}$ (cf. Figure 2, top panel)

(6) $$f_{B}=c_{Cd,T}^{gel}/c_{Cd,T}^{*}$$

Given knowledge of the concentrations $c_{Cd,T}^{gel}$ and $c_{Cd,T}^{*}$ from the electrochemical measurements performed along the lines given in Section 2.5, $f_{B}$ can be evaluated according to Equation (6). In turn, this value can be employed to estimate the metal partitioning between hydrogel and solution for different gel height (or volume) of the sample according to

(7) $$c_{Cd,T}^{gel}=c_{Cd,T}^{o}/\left[\varphi +\left(1-\varphi \right)/f_{B}\right]$$

which is obtained after combining Equation (6) with the mass balance equation.

By evaluating the *f*_B_ value applicable to the silica hydrogel system, we can accurately compute the total concentration of Cd that is accumulated in the hydrogel as a function of the (total) amount of Cd initially introduced in the sample. This value of *f*_B_ is further employed in the procedure we design below to refine our comparative analysis of the bioluminescent response of Cd-metal bioreporters in solution and in hydrogel scenarios.

### 3.4. Theoretical Formulation of the Bioluminescence Emission over Time: Case of Hydrogels-Embedded Biosensors

As detailed in Section 4.1, under all conditions examined in this work, the partitioning of Cd between the electrolyte solution and the hydrogel reaches equilibrium within a timescale (~16 min at most) that is much faster than the delay (~1–2 h) required to measure the onset of the bioluminescence response of the sensors over time. Accordingly, within the time window where metal-sensing cells are photoactive, there is no macroscopic Cd concentration gradient across the hydrogel and each cell in the hydrogel thus probes the same local concentration of bioavailable Cd ions, which is similar to the solution configuration examined in Section 3.3. The bioluminescence generated at time *t* by the assembly of biosensors distributed in the whole volume of the hydrogel can then be viewed as the integral average of the bioluminescence produced locally at the hydrogel altitude *z* with 0 ≤ *z* ≤ *h* (Figure 2). This integral average can be constructed from Equations (3)–(5) according to

(8) $$\mathrm{Lum}(t)=\theta_{gel}\sigma \frac{1}{h}\frac{K_{H}k_{int}}{1+{\left(K^{\prime }\right)}^{gel}}\;c_{B,T}^{\mathrm{gel}}\left(1-\gamma_{gel}{\;S}_{a}c_{B,T}^{\mathrm{gel}}\right)c_{Cd,T}^{gel}\int_{0}^{h}\left\{F\;\left(t\right)⊗\bar{c_{B,p}^{gel}}\left(t,z\right)\right\}dz$$

where $c_{B,T}^{\mathrm{gel}}$ and $c_{Cd,T}^{gel}\;$are defined in Section 3.1 (cf. Figure 2), σ is given by $\sigma =S_{a}{hS}_{gel}k_{\nu }k_{f}\tau_{q}K_{\mathrm{Hi}}^{-1}$ (in counts s^−1^ mol^−1^ m^5^) taken independent of *z*, $S_{gel}$ (m^2^) is the cross-sectional area of the hydrogel, $\gamma_{gel}$ (m) is the coefficient for passive adsorption of Cd at the surface of the biosensors in the hydrogel and $c_{Cd,T}^{O}$ relates to $c_{Cd,T}^{gel}$ (or $c_{Cd,T}^{*}$) via Equations (6) and (7). Equation (8) involves the dimensionless quantity $\theta_{gel}$ defined by $\theta_{gel}=c_{B,p}^{gel,max}/c_{B,T}^{\mathrm{gel}}$, which is for the hydrogel-embedded cells what θ defined in Section 3.2 is for cells in solution (cf. Equation (5)). Similar equivalence applies between the dimensionless cell photoactivity in the hydrogel, $\bar{c_{B,p}^{gel}}$, defined by $\bar{c_{B,p}^{gel}}=c_{B,p}^{gel}/c_{B,p}^{gel,max}$ and ${\bar{c}}_{B,p}$ involved in Equation (5).

For the sake of simplicity, Equation (8) tacitly implies that the variation of local bioluminescence along the *z*-direction at a given *t* originates from the *only* corresponding change in cell photoactivity $c_{B,p}^{gel}$ or $\bar{c_{B,p}^{gel}}$ due to the existence of a gradient of nutrients (e.g., O_2_) in the hydrogel phase. This is motivated by the strong connections existing between the time-dependent cell photoactivity, the quality and the quantity of resources required for cells to sustain light production [16,23,24]. We show that the form of Equation (8) proposed here for orientational purpose, is sufficient to identify and understand the ways the hydrogel matrix affects the time response of the biosensors distributed therein, in comparison to the situation where the biosensors are dispersed in an aqueous medium.

## 4. Results and Discussion

### 4.1. Cadmium Partitioning Between Hydrogel and Solution

#### 4.1.1. Time-Dependence of the Cadmium Concentration in the Hydrogels

An illustrative example of the time variation of metal concentrations measured in the hydrogel and in the outer electrolyte solution is shown in Figure S2 (SM-D) for a thick hydrogel layer (*h =* 6.0 ± 0.1 mm) at three solution ionic strengths (10, 30 and 100 mM). From these data, a diffusion coefficient of the metal species in the hydrogel phase can be evaluated by means of a simple diffusion approach using Crank’s model (Supporting Material, SM-D). The so-obtained diffusion coefficient in the silica hydrogel is (4.2 ± 2.0) × 10^−10^ m^2^ s^−1^ at 20 °C, which is 35% lower than that in water [47] (6.37 × 10^−10^ m^2^ s^−1^ at 20 °C) and quite similar to that reported for the diffusion of Cd(II) in an uncharged polymeric hydrogel like polyacrylamide [48] (4.02 × 10^−10^ m^2^ s^−1^ at 20 °C). It is stressed that the accelerating effect of the electrostatic field located at the hydrogel/solution interface on the diffusion of Cd species from solution to the hydrogel is here insignificant because the hydrogel dimension is orders of magnitude larger than the characteristic electrostatic Debye length [49]. This acceleration effect is further inexistent in the hydrogel as the potential therein is constant and equates to the Donnan value (the electrostatic field is thus 0 in the hydrogel). From the estimated diffusion coefficient, we can evaluate the time (from Equation (S3) in SM-D) needed to reach the equilibrium partitioning of Cd between the solution and hydrogel. For *h* varying from 675 µm to 1610 µm, this time does not exceed 1000 s (~16 min), meaning than the distribution of Cd over the whole volume of the hydrogel is achieved well before the biosensors start to produce bioluminescence (cf. the data discussed in Section 4.2.1).

#### 4.1.2. Boltzmann Cd Enrichment Factor in the Hydrogel, $f_{B}$

A fraction of the background electrolyte cations and total Cd species accumulated in the hydrogels are electrostatically associated with the negatively charged hydrogel components. The enrichment factor of Cd species in the hydrogel, $f_{B}$, is simply given by the ratio between the total Cd concentration in the gel over the metal concentration in external solution at equilibrium (Equation (6)). For situations where the hydrogel is in contact with the nGGM 1:5 solution adopted for the bioluminescence assays, we find $f_{B}$ = 5.8 ± 1.0, determined along the lines detailed in Section 3.3.

### 4.2. Analysis of Biosensor Responses to Cadmium Exposure

#### 4.2.1. Comparing the Time Dependence of Bioluminescence Cell Response in Solution and in Hydrogel

**Figure 3:**
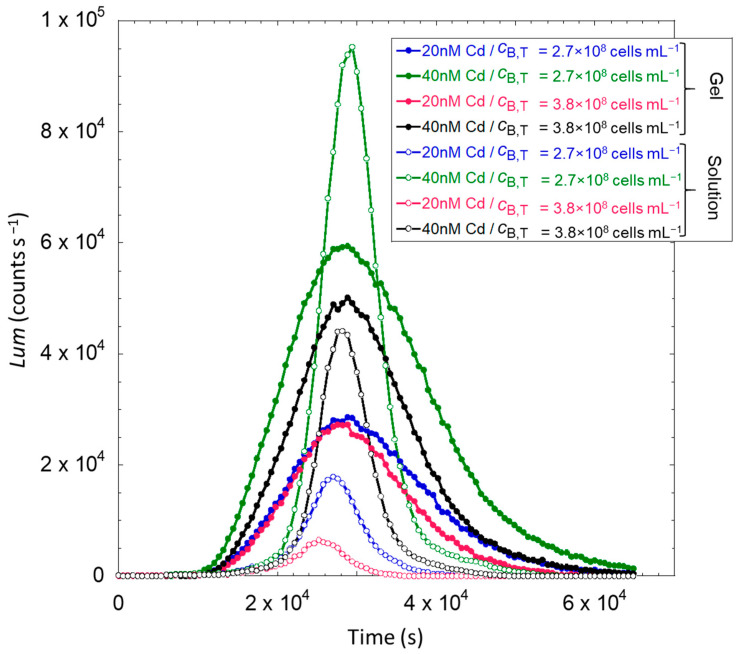
Time-dependence of the bioluminescence emitted by the Cd-inducible whole-cell bioreporters JW3596-L in silica hydrogels (plain circles) and in solution (open circles) for two selected values of total cadmium concentration $c_{Cd,T}^{O}$ = 20 nM and 40 nM (specified) and total cell concentration $c_{B,T}$ = 2.7 × 10^8^ cells mL^−1^ and 3.8 × 10^8^ cells mL^−1^ for gel thickness of 832 µm and 1143 µm, respectively (specified, cf. nomenclature in Figure 2). Experiments were performed in nGGM 1:5 medium at pH = 7.2.

The data set presented in Figure 3 depicts the time-dependence of the bioluminescence emitted by the Cd-inducible whole-cell bioreporters JW3596-L in silica hydrogels (plain circles) and in solution (open circles) for two selected values of total cadmium concentration $c_{Cd,T}^{O}$ = 20 nM and 40 nM (specified) and total cell concentration $c_{B,T}$ = 2.7 × 10^8^ cells mL^−1^ and 3.8 × 10^8^ cells mL^−1^ for gel thickness of 832 µm and 1143 µm, respectively (specified, cf. nomenclature in Figure 2). Experiments were performed in nGGM 1:5 medium at pH = 7.2 for free-floating biosensors and for biosensors immobilized in silica hydrogel, both exposed to Cd solutions prepared in nGGM 1:5. Here, we only report the results for two selected total Cd concentrations, $c_{Cd,T}^{O}$, of 20 nM and 40 nM, and for two total cell concentrations,$\;c_{B,T}$, of 2.7 × 10^8^ and 4.0 × 10^8^ cells mL^−1^ (cf. nomenclature in Figure 2). It is stressed that the concentration of biosensors in the hydrogel is constant and equates 1.71 × 10^9^ cells mL^−1^, and that $c_{B,T}$ is thus directly proportional to the gel to total volumes ratio according to $c_{B,T}=c_{B,T}^{\mathrm{gel}}V^{\;\mathrm{gel}}/{V\;}^{T}$.

Regardless of the dispersion medium of the biosensors, i.e., solution or hydrogel, the time dependence of their bioluminescence response follows a bell-shaped function with a maximum reached at a time, denoted as *t*_max_, between 2.6 × 10^4^ and 2.8 × 10^4^ s. Major differences between the two examined measurement configurations relate to the amplitude of the maxima (see details after in Section 4.2.3) and to the widths of the peaks. Namely, the bioluminescence maxima are higher for cells in solution and the time-window of the cell response is broader for hydrogel-embedded cells. Taking as an example the results obtained for $c_{Cd,T}^{O}=$ 40 nM and $c_{B,T}$ = 2.7 × 10^8^ cells mL^−1^, the peak width at mid-height is 0.73 × 10^4^ s for cells in solution and 2.0 × 10^4^ s for cells in the hydrogels.

These results reveal that the performance and ability of the biosensors to probe Cd are well-conserved when entrapped in the silica hydrogel. However, their response significantly differs over time from the one measured for the biosensors in solution. According to Duval et al. [16], the bioluminescence response pattern is strongly related to the dependence of the (dimensionless) concentration of photoactive cells on time, denoted as ${\bar{c}}_{B,p}$ and $\bar{c_{B,p}^{gel}}$ for the solution and hydrogel situations, respectively (Equations (5) and (8)). This concentration refers to the number of photoactive cells at *t*, with each of these cells having in common a constant bioluminescence yield. Equivalently, it corresponds to a time-dependent bioluminescence yield that applies to *all* cells present in the medium of interest [16,23,24]. As evidenced in [16] and elsewhere [18,24], cell photoactivity generally depends on time according to a sigmoid-like relationship, with an initial increase followed by a steeper growth before reaching a plateau value at a sufficiently long time *t*.

Back to our results, the broadening of the bioluminescence peak observed for the biosensors in the hydrogel then reflects a corresponding overall cell photoactivity that increases more gradually with time than that of the biosensors dispersed in solution. From inspection of Equation (8), this overall cell photoactivity at *t*, denoted as $\langle \bar{c_{B,p}^{gel}}\left(t\right)\rangle$, can be defined by

(9) $$\langle \bar{c_{B,p}^{gel}}\left(t\right)\rangle =\frac{1}{h}\int_{0}^{h}\bar{c_{B,p}^{gel}}\left(t,z\right)dz$$

Equation (9) suggests that the gradual increase of $\langle \bar{c_{B,p}^{gel}}\left(t\right)\rangle$ with *t* at the origin of the peak broadening is a consequence of the spatial distribution of the cell metabolic activity along the *z*-direction in the hydrogel. In contrast, this activity is probably more uniform from one cell to another when dispersed in solution. Remarkably, the position *t*_max_ of the bioluminescence maxima are identical for both planktonic and hydrogel-embedded biosensors. As demonstrated in [16,17], this property means that the inflection point associated with the sigmoid representing the time evolution of $\langle \bar{c_{B,p}^{gel}}\left(t\right)\rangle$ and ${\bar{c}}_{B,p}\left(t\right)$ are identical, whether biosensors are in solution or in the hydrogel.

The above differences between $\langle \bar{c_{B,p}^{gel}}\left(t\right)\rangle$ and ${\bar{c}}_{B,p}\left(t\right)$ may be explained a priori by a change of the bioavailability properties of Cd ions and/or nutrients depending on the altitude *z* in the hydrogel. The time required for the Cd distribution to reach equilibrium between solution and hydrogel is much shorter (~16 min for the thicker hydrogel, cf. Section 4.1) than the delay marking the onset of bioluminescence response over time (1 h for cells in hydrogel and 1.5 h in solution, Figure 3). Accordingly, as already mentioned in Section 3.4, each cell in the hydrogel is expected to probe the same local concentration of Cd ions. In turn, this leaves us with the hypothesis that $\langle \bar{c_{B,p}^{gel}}\left(t\right)\rangle$ and ${\bar{c}}_{B,p}\left(t\right)$ differ as a result of a change in the bioavailability of the nutrients depending on the location *z* of the biosensors in the hydrogel. This issue is specifically examined in the next section with the use of constitutive *lux*-biosensors designed to monitor cell activity.

#### 4.2.2. Time-Dependent Response of Constitutive Lux-Biosensors in the Hydrogel and Solution Measurement Configurations

The analysis of the responses of the *rrnB* P1-*luxCDABE* biosensor (JW3596-C) provides valuable information on the sequence of nutrients regulation mechanisms adopted by the cells, regardless of Cd bioavailability [18]. These biosensors constitutively produce bioluminescence as a result of the expression of the *lux*-reporter genes placed under the transcriptional control of the ribosomal RNA *rrnB* P1 promoter. The transcription is repressed in the presence of the alarmone (p)ppGpp synthetized by the bacteria under nutrient-limited conditions. The alarmone production triggers the so-called stringent response, which defines a transitory period between the consumption of the entire pool of extracellular amino acids by the cells and the end of their intracellular production of amino acids. The interaction of the alarmone with the RNA polymerase immediately turns off the ribosomal RNA synthesis prioritizing the cellular resources for the biosynthesis of amino acids. Once the amino acid levels are restored, the cells recover their ability to produce bioluminescence. As evidenced in Delatour et al. [18], the time response of JW3596-C, hereafter denoted as ${\mathrm{Lum}}_{c}\left(t\right)$ (with ‘c’ standing for constitutive), mirrors the time evolution of the cell photoactivity.

**Figure 4:**
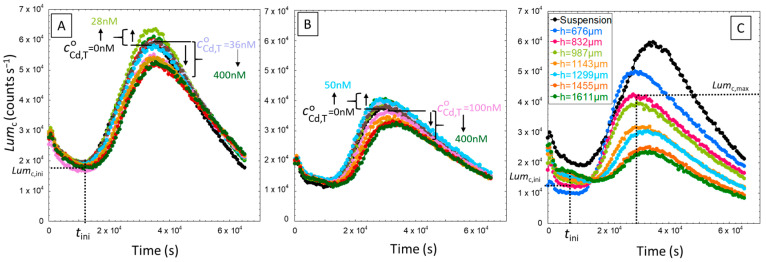
Time-dependent bioluminescence response of constitutive whole-cell reporters JW3596-C dispersed in aqueous media (**A**), or embedded in the silica hydrogel (h = 987 µm) (**B**), for a total cadmium concentration $c_{\mathrm{Cd},T}^{\;O}$ ranging from 0 nM to 400 nM (specified). Time-dependent response of constitutive whole cell reporters JW3596-C embedded in hydrogels with thickness h between 0 (the solution configuration, referred to as ‘suspension’ case in the caption) to 1611 μm (specified) for $c_{\mathrm{Cd},T}^{\;O}$ = 20 nM (**C**). Experiments were performed in nGGM 1:5 medium at pH = 7.2.

We report in Figure 4A,B the response ${\mathrm{Lum}}_{c}\left(t\right)$ of constitutive biosensors in solution and silica hydrogel measurement configurations for a hydrogel thickness *h* = 987 µm in a nGGM medium (1:5 dilution) and a total Cd concentration $c_{Cd,T}^{O}$ in the range of 0–400 nM. Figure 4C further shows ${\mathrm{Lum}}_{c}\left(t\right)$ measured for $c_{Cd,T}^{O}$ = 20 nM and a hydrogel thickness varied between 0 (this is the solution measurement configuration limit) and ~1.6 mm. In the absence of hydrogel (Figure 4A), ${\mathrm{Lum}}_{c}\left(t\right)$ slightly differs from the results of Duval et al. [17] who measured a constant bioluminescence in the early stage of the response, between 1 and 2 × 10^4^ s, in the same nutritive medium (nGGM 1:5) as that employed here. This initial bioluminescence emission regime referred to the stringent cell response due to a deficiency of amino acids in the medium. Instead, under the conditions examined here a decrease of bioluminescence is measured till 1.5 × 10^4^ s. This difference may arise from variation in the experimental preconditioning of the cells prior to bioluminescence measurements. In our work, hydrogels and suspensions containing bacteria were kept at 4 °C for 3 h before exposure to metal-containing medium. For the situation where cells were dispersed in solution, biosensors were maintained in nGGM medium prior to dilution and bioluminescence measurements in the presence of Cd added at *t* = 0. This preconditioning of the biosensors in poorly nutritive media may modify cell metabolism and in turn impact on the initial bioluminescence production as compared to that reported in Duval et al. [17]. Once this initial stage is passed, i.e., for *t >* 2 × 10^4^ s when the transitory stringent response is completed, bioluminescence increases and reaches a maximum value, denoted as ${\mathrm{Lum}}_{c,\max},$ at *t ~* 4 × 10^4^ s. Then, it decreases quasi-linearly with time, in agreement with the results in [17]. Changes in the Cd concentration $c_{Cd,T}^{O}$ slightly impact the magnitude of the bioluminescence maxima. Namely, the curves ${\mathrm{Lum}}_{c}\left(t\right)$ measured for $c_{Cd,T}^{O}$ below 28 nM are confounded with or slightly above the one corresponding to $c_{Cd,T}^{O}=$0 nM, whereas ${\mathrm{Lum}}_{c,\max}$ was found to decrease with increasing concentration above 28 nM. This finding agrees with the results obtained in [17] and the occurrence of hormesis effects at sufficiently low concentrations of Cd.

**Figure 5:**
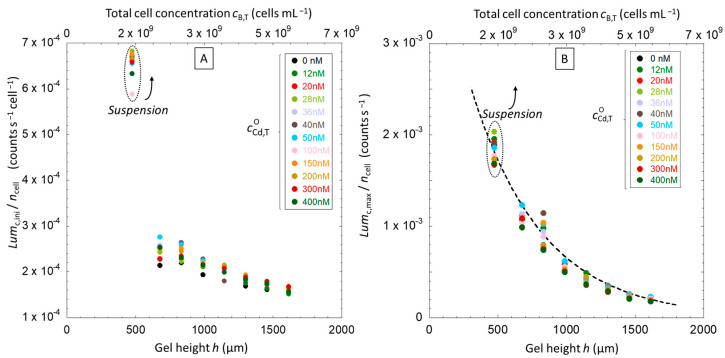
Characteristic values of the bioluminescence per cell, ${\mathrm{Lum}}_{c,\mathrm{ini}}/n_{\mathrm{cell}}$ (**A**) and ${\mathrm{Lum}}_{c,max}/n_{\mathrm{cell}}$ (**B**), versus the total cell concentration, $c_{B,T}$, or the hydrogel height h (relevant for hydrogel-embedded-cells only) for different values of the total cadmium concentration, $c_{\mathrm{Cd},T}^{O}$, ranging between 0 and 400 nM (indicated). The quantities ${\mathrm{Lum}}_{c,\mathrm{ini}}$ and ${\mathrm{Lum}}_{c,max}$ refer to the two regimes defined in Figure 4C for the time response of the whole-cell constitutive reporters JW3596-C. Experiments were performed in nGGM 1:5 at pH = 7.2. In panel (**B**), the dotted line represents the exponential decay function that fits the dependence of ${\mathrm{Lum}}_{c,\max}/n_{\mathrm{cell}}\;$on $c_{B,T}$.

Let us now compare the emission of bioluminescence over time for the two types of bioluminescence measurement configurations, i.e., in solution and hydrogel (Figure 4B,C). In both configurations, the position and general features of the cell response are very similar and two distinct regimes can be distinguished: an initial phase of bioluminescence emission from *t* = 0 to 1 × 10^4^ s corresponding to a decrease of light production with time and the achievement of a constant basal light emission level, followed by a stronger light production after completion of the cell stringent response. Two characteristic values of the bioluminescence response are defined to describe these regimes: ${\mathrm{Lum}}_{c,\mathrm{ini}}$ which refers to the basal level of light emission, and ${\mathrm{Lum}}_{c,max}$ which stands for the maximum of bioluminescence reached in the second emission regime (Figure 4C). For hydrogel embedded-cells, ${\mathrm{Lum}}_{c,\mathrm{ini}}$ is reached at *t ~ *3–8 × 10^3^ s whereas for cells in solution, the plateau is less marked and we chose to evaluate ${\mathrm{Lum}}_{c,\mathrm{ini}}$ at *t* = 1 × 10^4^ s. To facilitate the comparison between the hydrogel and the solution configurations over the whole range of Cd concentrations and total cell concentrations tested, we report in Figure 5 the corresponding values ${\mathrm{Lum}}_{c,\mathrm{ini}}/n_{\mathrm{cell}}$ and ${\mathrm{Lum}}_{c,max}/n_{\mathrm{cell}}$, where $n_{\mathrm{cell}}$ is the total amount of cells in the volume where biosensors are distributed, i.e., the solution for the case of planktonic cells and the hydrogel for hydrogel-embedded cells.

According to Figure 5A, the bioluminescence ${\mathrm{Lum}}_{c,\mathrm{ini}}/n_{\mathrm{cell}}$ produced per cell decreases with the gel layer thickness *h* according to a linear relationship. This trend suggests a possible loss of nutrients availability and/or oxygen uptake when increasing the number of cells in the sample or, equivalently, when increasing the hydrogel thickness. At the time selected to evaluate ${\mathrm{Lum}}_{c,\mathrm{ini}}/n_{\mathrm{cell}}$, i.e., prior to the increase of bioluminescence in the second emission regime identified in Figure 4C, there is a deficiency of amino acids in the medium, and the resulting nutrient deprivation is likely to be amplified when increasing the number of cells in the sample. Moreover, ${\mathrm{Lum}}_{c,\mathrm{ini}}$ measured in the hydrogel is much lower than that measured in the solution case, thus underlining that the bioavailability of resources, including oxygen, is strongly reduced as compared to that for the suspension case. It is worth stressing that at a given hydrogel height *h*, ${\mathrm{Lum}}_{c,\mathrm{ini}}/n_{\mathrm{cell}}$ in the presence of Cd is always equal or higher than values obtained without metal (black points). This confirms the hormesis effects detected in Figure 4, even though the Cd concentration in the hydrogel, $c_{\mathrm{Cd},T}^{\mathrm{gel}}$, is four to five times larger than the total Cd concentration applying to the solution situation, $c_{Cd,T}^{O}$, due to the electrostatic effects materialized by the coefficient $f_{B}$ computed in Section 4.1.2.

Figure 5B evidences that the bioluminescence production per cell in the second emission regime defined in Figure 4C decreases with increasing *h*, for reasons similar to those invoked in Figure 5A to explain the decrease of ${\mathrm{Lum}}_{c,\mathrm{ini}}/n_{\mathrm{cell}}$ with the hydrogel height. The difference here is that ${\mathrm{Lum}}_{c,max}/n_{\mathrm{cell}}$ decreases with *h* according to an exponential law (cf. dotted lines in Figure 5B). Most remarkably, we note that this exponential fitting function well describes not only the data pertaining to the situation of hydrogel-embedded cells but also the data points corresponding to the case where cells are dispersed in solution in the absence of hydrogel. We further emphasize that, by construction, this exponential function mirrors the cell photoactivity function $\langle \bar{c_{B,p}^{gel}}\left(t\right)\rangle$ (Equation (9)) taken here at *t* = *t*_max_ [18].

This exponential-like decrease of the cell photoactivity with thickness *h* recalls the typical oxygen concentration profiles reported in porous media like sediments [50], hydrogels [51,52,53] or tissue materials [54]. Such profiles reflect the relationship between oxygen consumption by organisms and the diffusion properties of oxygen across complex 3D environmental matrix. Concentrations of O_2_ are typically largest at the surface of these matrices or in the direct vicinity of O_2_ sources, while O_2_ is gradually consumed across the matrices as a result of microbial activity therein, resulting in a decrease of oxygen concentration with depth or distance from the oxygen source. Our experiments suggest that an increase of hydrogel thickness leads to oxygen-limited environments, which in turn reduces the maximum production of bioluminescence per cell, ${\mathrm{Lum}}_{c,max}/n_{\mathrm{cell}}$ (Figure 5B) and also the kinetics of light production. The latter finding is evidenced by the drop with increasing *h*- of the slope of the linear increase ${\mathrm{Lum}}_{c}\left(t\right)$ *versus* time in the time window that precedes the maxima (Figure 4C). It can be concluded from Figure 4 and Figure 5 that cell photoactivity most likely decreases from the top to the bottom part of the hydrogel according to an exponential decay function (Equation (9), with a bioavailability of resources and/or O_2_ that is largest for cells located near the interface formed with the outer solution. The resulting amplitude and rate of bioluminescence emission by these top cells in the hydrogel are larger as compared to cells distributed deeper in the hydrogel. As mentioned earlier, this heterogeneity likely explains why Cd-inducible biosensors embedded in the hydrogel generate a bioluminescence response that spans over a time-window broader than that of biosensors dispersed in solution (Figure 3).

#### 4.2.3. Response of Cd-Inducible Lux-Biosensors in Solution and Hydrogel as a Function of Total Cell Concentration

**Figure 6:**
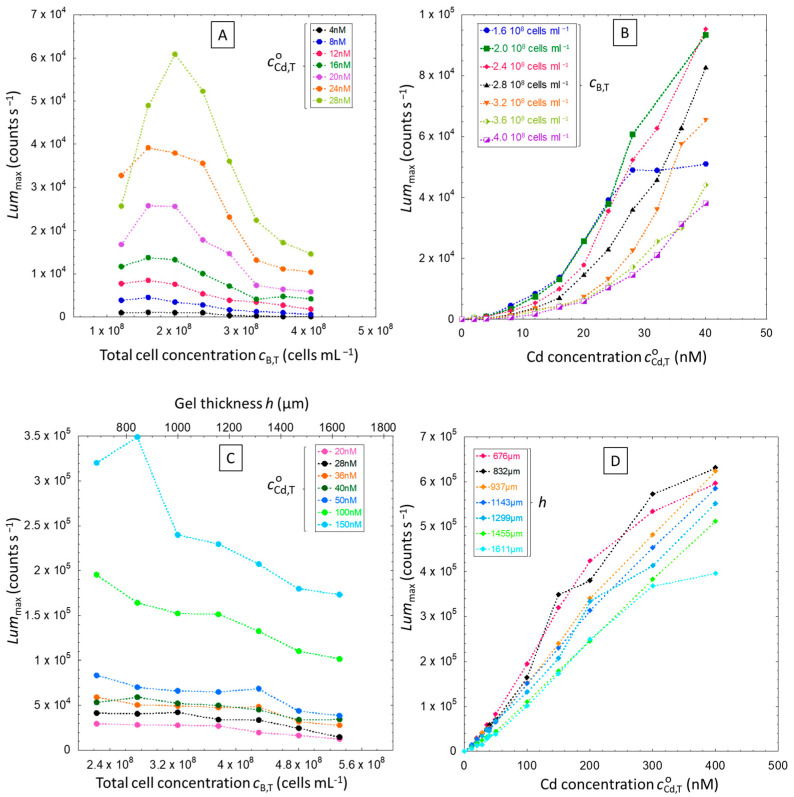
Dependence of ${\mathrm{Lum}}_{max}$ on total cell concentration, $c_{B,T}$, for JW3596-L biosensors in solution at various total metal concentrations, $c_{Cd,T}^{O}$ (indicated) (**A**), and on total Cd concentration, $c_{Cd,T}^{O}$, at various cell concentrations, $c_{B,T}$ (indicated) (**B**). Dependence of ${\mathrm{Lum}}_{\max}$ on total cell concentration, $c_{B,T}$, for JW3596-L biosensors embedded in silica hydrogels at various total cadmium concentrations, $c_{Cd,T}^{O}$ (indicated) (**C**), and on total cadmium concentration in the sample, $c_{Cd,T}^{O}$, for various hydrogel thickness, *h* (indicated) (**D**). The results were obtained in nGGM 1:5 and pH 7.2. Dotted lines are guides to the eye.

We first proceed to the discussion of the results obtained for Cd-inducible biosensors dispersed in solution, with the bioluminescence maxima ${\mathrm{Lum}}_{\max}$ reported in Figure 6 for various total cell concentrations $c_{B,T}$ (0.8 × 10^8^ to 4.0 × 10^8^ cells mL^−1^) and total Cd concentration $c_{Cd,T}^{O}$ (0 to 40 nM).

Pagnout et al. [15] have reported a quadratic-like relationship (concave down parabola) between maximal rate of emitted bioluminescence and total cell concentration at a given total bulk metal concentration. In line with theory [15,16], this relationship reflects the balance between the amount of internalized metal ions and the amount of metal adsorbed at sites of the cell membrane that do *not* lead to internalization (so-called passive metal adsorption). Increasing cell density first leads to an increase of the bioluminescence produced all over the solution volume. For cell densities exceeding a given threshold value, the overall cell surface area in solution becomes so large that the bioluminescence decreases due to significant passive surface adsorption of the metals and to resulting reduction in metal bioavailability in solution. This pattern is well reproduced in Figure 6A, with the values of ${\mathrm{Lum}}_{\max}$ plotted as a function of $c_{B,T}$. In line with [15], the larger $c_{Cd,T}^{O}$ is, the more pronounced becomes the maximum of ${\mathrm{Lum}}_{\max}$ versus $c_{B,T}$. Figure 6B displays the data of Figure 6A in such a way that the dependence of ${\mathrm{Lum}}_{\max}$ on $c_{Cd,T}^{O}$ is now illustrated for different values of $c_{B,T}$. The linear part of these curves reached for sufficiently large values of $c_{Cd,T}^{O}$ corresponds to the so-called linear Henry regime of Cd(II) adsorption on the internalization sites. For the lowest tested value of the total cell concentration,$\;c_{B,T}$ = 1.6 × 10^8^ cells mL^−1^, the linear Henry regime is restricted to the total Cd concentration ranging from ca. 20 nM to 30 nM. At such a low cell concentration level, ${\mathrm{Lum}}_{\max}$ reached at sufficiently large Cd concentration reaches a constant value. Given that a significant loss of bioluminescence is observed for lower values of $c_{B,T}$ (e.g., $c_{B,T}=$1.2 × 10^8^ cells mL^−1^) over the whole range of Cd concentration, we assign this behavior to Cd-mediated toxicity that becomes operational at sufficiently low cell-to-metal concentration ratios.

Let us now examine the counterpart of Figure 6A,B for hydrogel-embedded cells (Figure 6C,D).

Unlike Figure 6A, Figure 6C does not show a quadratic dependence of ${\mathrm{Lum}}_{\max}$ on $c_{B,T}$ but, instead, a rather constant ${\mathrm{Lum}}_{\max}$ except for the two largest total Cd concentrations tested (100 and 150 nM). This finding may be the result of several combined features that are not compatible with the observation of a bell-shaped ${\mathrm{Lum}}_{\max}$ vs. $c_{B,T}$ curve as measured for cells in solution. First, we saw in Figure 5B that the overall photoactivity of constitutive biosensors in the hydrogel decreases when the hydrogel thickness (or, equivalently, $c_{B,T}^{\mathrm{gel}}$) increases, i.e., there is an overall reduction of light production in the hydrogel, and more so as the hydrogel thickness increases. Moreover, both passive metal adsorption at the cell membrane and metal complexation in the hydrogel are operative, which decreases the metal bioavailability and in turn the bioluminescence production. In other words, the range of bioavailable metal-to-cell ratios relevant for the hydrogel situation is significantly lower than that pertaining to the solution scenario. At this stage, it then becomes already clear that the speciation and the local concentration of metal species are different in the hydrogel and solution scenarios. For a given hydrogel thickness, we observed a linear dependence of ${\mathrm{Lum}}_{\max}$ on the total Cd concentration, $c_{Cd,T}^{O},$ comparable to what we measured for cells in solution (Figure 6D). However, here the range of Cd concentration where linearity applies is significantly larger, from 0 nM to 400 mM.

#### 4.2.4. Comparison Between Bioluminescence Emitted per Cell in Relation and Total Amount of Cd per Cell in the Solution and Hydrogel Scenarios

**Figure 7:**
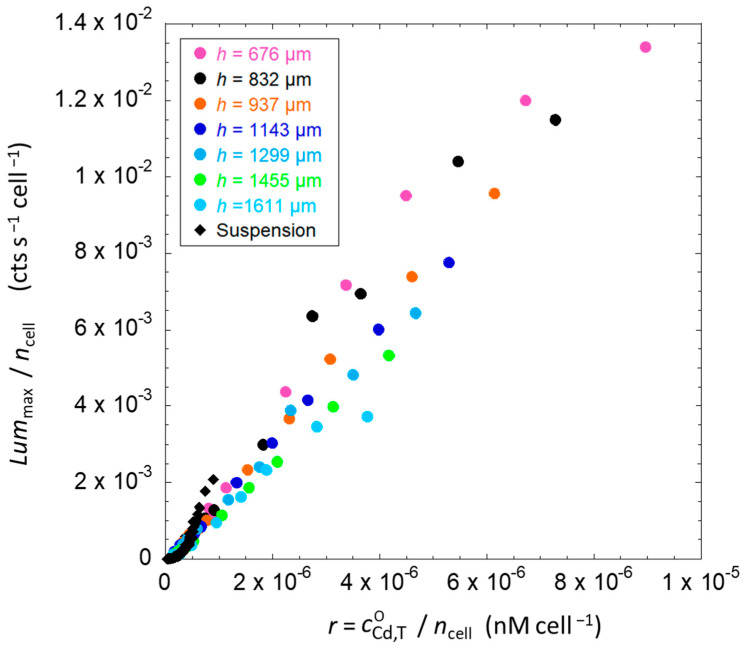
Values of ${\mathrm{Lum}}_{max}/n_{\mathrm{cell}}$ as a function of ${r=c}_{Cd,T}^{O}/n_{\mathrm{cell}}$ for JW3596-L cells embedded in hydrogels of different thickness *h* (indicated, colored dots) or dispersed in solution (suspension case, black diamonds, indicated). The data were measured in nGGM 1:5 at pH 7.2.

In the following, a unified interpretation scheme is detailed for the data given in Figure 6 that pertain to the two distinct solution and hydrogel measurement configurations of interest in this work. The procedure we adopted to perform this interpretation consists first in representing the maxima of bioluminescence produced per cell, i.e., ${\mathrm{Lum}}_{max}/n_{\mathrm{cell}}$, as a function of the ratio *r* between total Cd concentration and total amount of cells, i.e., ${r=c}_{Cd,T}^{O}/n_{\mathrm{cell}}\;$(Figure 7). For the solution case, the ratio *r* is calculated from bulk parameters, i.e., the total cadmium concentration in solution $c_{Cd,T}^{o}$ and the amount of cells, by converting the optical density into a number of bacteria in the solution volume. For the gel case, as a first approximation, *r* is evaluated from the ratio between the total Cd concentration over the whole volume $c_{Cd,T}^{o}$, and the total number of bacteria introduced in the sample. The results are reported in Figure 7.

Figure 7 shows that all data reported in Figure 6B for Cd-inducible biosensors in solution all collapse onto a single curve when represented according to a ${\mathrm{Lum}}_{max}/n_{\mathrm{cell}}$ *versus* ${r=c}_{Cd,T}^{O}/n_{\mathrm{cell}}$ representation (also see the linear-log representation of the data in Figure S3 of the Supporting Material, SM-E). This suggests the existence of a direct relationship between the amount of Cd concentration per cell and the level of bioluminescence produced per cell, regardless of the initial amount of cells in the sample. A similar per-cell adjustment of the data given in Figure 6C,D for hydrogel-embedded cells also leads to a strong reduction of the original data dispersion featured by, for example, Figure 6D. Still, the applied scaling fails to generate a single curve where all hydrogel-related data would fall as there clearly remains some scattering between data obtained under different hydrogel thickness conditions (Figure 7). Within the procedure we followed to generate Figure 7, the relevant concentration of Cd in the hydrogel is taken to be identical to that in the solution phase above the hydrogel, i.e., $c_{Cd,T}^{o}$~$c_{Cd,T}^{gel}$. This latter equality, however, ignores the accumulation of Cd in the hydrogel due to electrostatic effects (cf. Section 3.3). As explained in Section 3, the total concentration of Cd(II) in the hydrogel volume can be evaluated from Equation (6) using the Boltzmann partitioning coefficient $f_{B}$ that we determined from the electroanalytical measurements detailed in Section 4.1.2. In turn, we can therefore convert the ratio *r* = $c_{Cd,T}^{o}/n_{cell}$ into its relevant equivalent *r’* = $c_{Cd,T}^{gel}/n_{\mathrm{cell}}$ for hydrogels using Equation (7), as we know the introduced Cd concentration ($c_{Cd,T}^{o}$) and gel volume fraction φ. The corresponding outcome in terms of data dispersion reduction is detailed in the next section.

#### 4.2.5. Comparative Analysis of the Bioluminescence Emitted per Cell in the Solution and Hydrogel Scenarios with the Account of Electrostatics-Mediated Cd Accumulation in the Hydrogel

**Figure 8:**
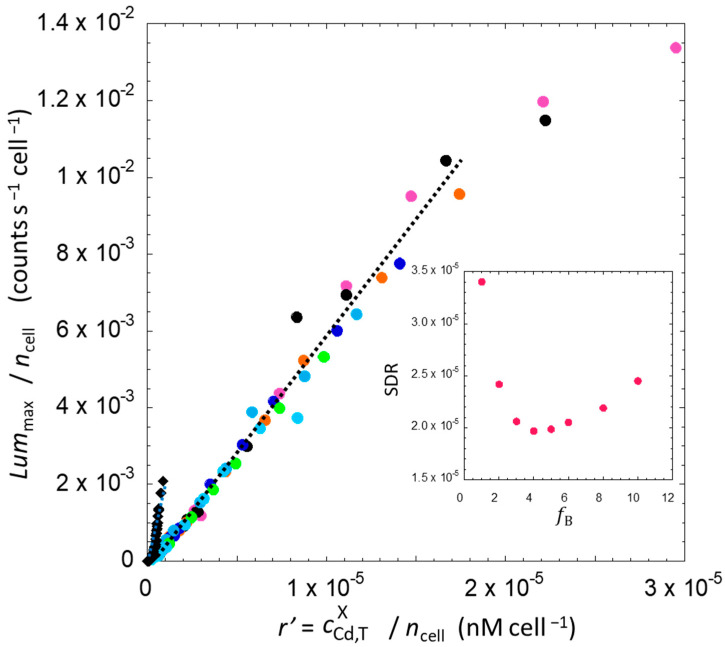
${\mathrm{Lum}}_{max}/n_{\mathrm{cell}}$ as a function of ${r^{\prime }=c}_{Cd,T}^{x=gel,sol}/n_{\mathrm{cell}}$ for JW3596-L biosensors cells in hydrogels with varied thickness (colored dots, specified) or in solution (black diamonds, indicated) with the account of electrostatic Cd accumulation using $f_{B}=5$. The plot collects all the data obtained for the hydrogel and solution measurement configurations. Data were measured in nGGM 1:5 at pH 7.2. The dotted lines represent the lines used to calculate the slopes of the linear sections (see Section 4.2.6). The inset represents the standard deviation of the residuals (SDR) calculated from the linear regressions of$\;{\mathrm{Lum}}_{max}/n_{\mathrm{cell}}\;$vs. $r^{\prime }=c_{Cd,T}^{gel}/n_{cell}$ at the different tested values of $f_{B}$.

To correct the data in Figure 7 for electrostatic Cd accumulation in the hydrogel, we proceeded as follows. The ratio *r* = $c_{Cd,T}^{o}/n_{\mathrm{cell}}$ was changed to *r’* = $c_{Cd,T}^{gel}/n_{cell}$ (see Equation (7) without a priori assumption on the value of $f_{B}$, and we adjusted $f_{B}$ from $f_{B}$ = 1 (no electrostatic effects) to 10 so as to obtain the best single master curve where all bioluminescence data collected in hydrogels collapsed with minimum data scattering. For each set of $f_{B}$-corrected ratio, we calculated the linear regression of all resulting (Y; X) data $\left({\mathrm{Lum}}_{max}/n_{\mathrm{cell}};r^{\prime }=c_{Cd,T}^{gel}/n_{cell}\right)$, together with the standard deviation of the residuals (SDR). The inset in Figure 8 illustrates the dependence of the SDR as a function of the adopted $f_{B}$ value. We found a remarkable merging of all data into a single master curve with a $f_{B}$ parameter in the range 4 to 5, values for which we obtained a minimum in SDR. This range of $f_{B}$-values is in excellent agreement with the value $f_{B}$ = 5.8 ± 1.0 independently determined by our Cd partitioning analysis with the help of the SCP electroanalytical technique (Section 4.1.2). The final result is given in Figure 8. Overall, Figure 8 makes it clear that the overall biosensor response measured in hydrogels is intimately connected to the concentration of Cd in the direct vicinity and local environment of each metal sensing cell. A similar conclusion holds for bioluminescent cells in solution.

Figure 8 shows that the ${\mathrm{Lum}}_{max}/n_{\mathrm{cell}}$ data pertaining to hydrogel-embedded cells are now shifted towards higher values of *r’* as compared to those relevant for the biosensors dispersed in solution (also see the linear-log representation of the data in Figure S4 of the Supporting Material, SM-E). This result is according to expectation as the total Cd concentration in the hydrogel is necessarily higher than that in the bulk solution (at equilibrium). Accordingly, there is some discrepancy between the two bioluminescence data sets at fixed *r*, which raises the question about the respective bioavailability of Cd ions in the hydrogel and in the solution. Our first hypothesis is that there is a fraction of Cd species bound to the reactive sites of the silica hydrogel and to the passive adsorption sites of the cell membrane, which lowers the Cd amount detectable by the biosensors. At this stage of the discussion, Cd speciation should be corrected on the basis of the theoretical developments discussed in Section 3. Such a correction is elaborated in the next section.

#### 4.2.6. Comparing the Bioluminescence Emitted per Cell Versus Free Cd Concentration per Cell in the Solution and Hydrogel Scenarios

The bioluminescence signal collected in the hydrogel scenario is formulated according to Equation (8). The latter equation, when applied at $t=t_{max}$, shows that ${\mathrm{Lum}}_{max}/n_{\mathrm{cell}}$ is proportional to $c_{Cd,T}^{gel}$ and that the proportionality coefficient involves the factor $\frac{1}{1+{\left(K^{\prime }\right)}^{gel}}$ where ${\left(K^{\prime }\right)}^{gel}$ is the dimensionless stability constant for the metal complexes formed in the hydrogel volume. Similarly, Equation (5) evidences that ${\mathrm{Lum}}_{max}/n_{\mathrm{cell}}$ evaluated for biosensors dispersed in solution depends linearly on $c_{Cd,T}^{sol}$ with a slope whose expression includes the term $\frac{1}{1+{\left(K^{\prime }\right)}^{sol}}$. Accordingly, for both hydrogel and solution measurement configurations, the slope of the line ${\mathrm{Lum}}_{max}/n_{\mathrm{cell}}$ vs. *r’* ratio, denoted as ${SLOPE}^{x=sol,gel}$, is proportional to 1/(1 + ${\left(K^{\prime }\right)}^{x=sol,gel}$). Assuming that all other factors defining ${SLOPE}^{x=sol,gel}$ are identical for the solution and hydrogel situations, in particular $\gamma_{gel}=\gamma$ (cf. Equations (5) and (8)), we may then write

(10) $$\frac{{SLOPE}^{sol}\;}{{SLOPE}^{gel}}=\frac{\left(1+{\left(K^{\prime }\right)}^{\mathrm{gel}}\right)}{\left(1+{\left(K^{\prime }\right)}^{\mathrm{sol}}\right)}$$

**Figure 9:**
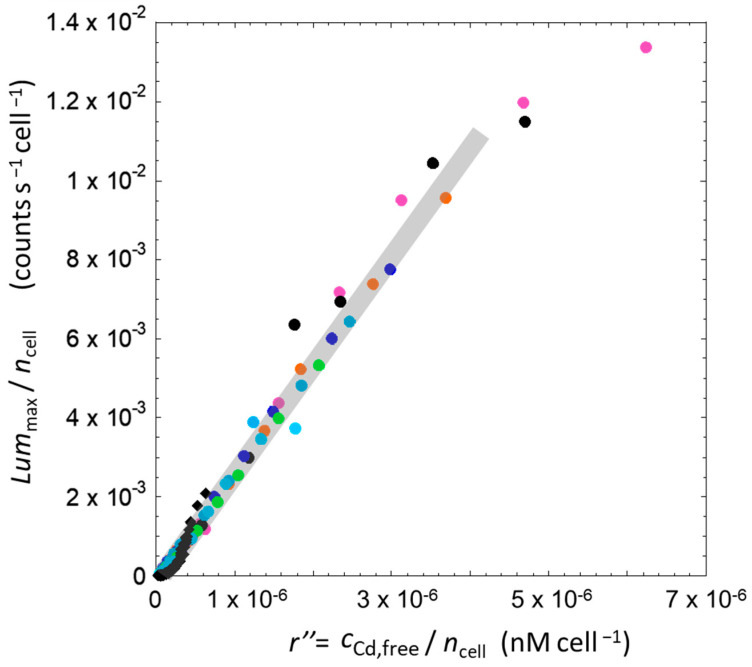
As in Figure 8, except that ${\mathrm{Lum}}_{max}/n_{\mathrm{cell}}$ data are reported here as a function of *r*″ = $c_{\mathrm{Cd},\mathrm{free}}/n_{\mathrm{cell}}$. The grey shaded area encompassing all data points is a guide to the eye.

Our purpose is then to evaluate ${\left(K^{\prime }\right)}^{gel}$ from Equation (10), recalling that the value of $\frac{{SLOPE}^{sol}\;}{{SLOPE}^{gel}}$ is obtained from the linear regimes of the bioluminescence data displayed in Figure 9 (with the result $\frac{{SLOPE}^{sol}\;}{{SLOPE}^{gel}}=3.9).$ To do so, we applied the following strategy. For the solution case, the Cd equilibrium speciation was evaluated using the electroanalytical technique called AGNES (Absence of Gradient and Nernstian Equilibrium Stripping), known to be a robust methodology to determine nanomolar level of free metal ions in solution (cf. details in SM-B) [55,56]. In nGGM 1:5 medium, we estimated that approximately 70% of Cd was in its free form, resulting in a ${\left(K^{\prime }\right)}^{sol}$ value of 0.42 ± 0.02. Then, using Equation (10), the value determined above for $\frac{{SLOPE}^{sol}\;}{{SLOPE}^{gel}}$ and a range of ${SLOPE}^{gel}$ that correctly bracket all the data in Figure 8, we estimated that ${\left(K^{\prime }\right)}^{gel}$ was equal to 4.5. We stress that the free Cd concentrations in solution and in the hydrogel can be computed from $c_{Cd,T}^{x=gel,sol}$ and $K^{\prime }$ using Equation (1). This indicates that only 18% of Cd is in free form and 82% is bound to the reactive silica sites or is passively adsorbed at the membrane of the biosensors. In turn, we can replot all data of Figure 8 by replacing the ratio *r’* therein by *r*″ defined as the ratio between free metal concentration ($c_{\mathrm{Cd},\mathrm{free}})$ and number of cells in the sample ($n_{\mathrm{cell}})$ (either hydrogel or solution) (Figure 9). The two data sets relevant for the two bioluminescence measurement configurations are now rescaled according to the same range of *r*″ values that refer to the bioavailable free metal concentration in the local environment of the biosensors. Figure 9 shows that the data sets originally given in Figure 6 (and obtained for cells in hydrogels of various thickness and in solution at different total cell concentrations and total Cd concentrations) all properly fall within *a single master curve* (also see the linear-log representation of the data in Figure S5 of the SM-E). The successive adjustments made to normalize the bioluminescence data collected in the solution and hydrogel scenarios address the paramount importance of electrostatics and operating metal speciation conditions, both having an impact on the amount of bioavailable free Cd concentration probed by the biosensors in their local microenvironment. Namely, our successful interpretation of the bioluminescence data measured in solution and hydrogel calls for a transition from an argumentation based on the total (initial) Cd concentration in the sample (quantity *r*) to an interpretative scheme that involves the total Cd concentration operational in either the solution or in the hydrogel (quantity *r*′) and the free Cd concentrations relevant in these two systems (quantity *r*″).

To gain a complete comprehensive understanding of the data, it is also necessary to address the contribution of cell photoactivity on the level of bioluminescence produced per cell for the two scenarios, solution and hydrogel, as discussed in the Section 4.2.2. The successful construction of the master curve displayed in Figure 9 following the procedure we detailed above suggests that any additional data adjustment is unlikely to generate a significant effect on the results. This expectation is qualitatively confirmed by comparing the dependence of the ratios ${\mathrm{Lum}}_{max}/n_{\mathrm{cell}}$ and ${\mathrm{Lum}}_{c,max}/n_{\mathrm{cell}}$ on the total cell number $n_{\mathrm{cell}}$ (Figure S6 in Supporting Material, SM-F). Briefly, the analysis demonstrates that the magnitude of the loss in cell photoactivity (decreasing ${\mathrm{Lum}}_{c,max}$) with increasing $n_{\mathrm{cell}}$ (or equivalently *h*) is not significant at all in comparison with the decrease in ${\mathrm{Lum}}_{max}$. Accordingly, the biosensing activity in the hydrogel appears to be largely driven by the chemical (bio)availability of the metal species, which is controlled by passive metal sorption on the cell surface and binding at the gel reactive sites, as well as by the electrostatic accumulation of Cd species within the gel.

Finally, there is a last remarkable implication of biosensor entrapment in the hydrogels that we need to comment on, and it relates to the range of Cd concentrations probed by the hydrogel-embedded biosensors that is significantly higher than the one sensed for the free-floating bacteria. The upper limit of the linear regime displayed in Figure 9 is indeed positioned at *r*″ = 7 × 10^−7^ nM cell^−1^ in the solution scenario and *r*″ = 4 × 10^−6^ nM cell^−1^ in the hydrogel case. It is recalled here that those data refer to the rate of photon emission per cell for a fixed amount of bioavailable free Cd. Given the higher cell photoactivity in solution than the one in the hydrogel, Cd uptake flux and efficiency of conversion into light are superior in the solution scenario. Consequently, from an energy standpoint, the bacteria dispersed in solution will probably deplete their energy resources at a higher rate than bacteria in hydrogels would do. In addition, it is expected that this effect will be more pronounced as the metal concentration increases. The metabolic demands by the bacteria in solution may result in their reduced capability to withstand elevated metal concentrations and related adverse effects. Conversely, the biosensors embedded in the hydrogels where there is a lower amount of available resources may be more efficient in maintaining a balance between metabolic activity dedicated to light production and other cell energy demands, which could explain the larger range of *r*″ values the biosensors can probe without experiencing toxicity effects.

## 5. Conclusions

This study presents a comprehensive investigation of the bioluminescence response of Cd(II)-inducible *PzntA-luxCDABE Escherichia coli* biosensors embedded in silica-based hydrogels. These matrices are viewed here as model systems that share representative properties with more complex confined environments, i.e., limited resource renewal and a possible accumulation of potentially harmful metals due to intrinsic matrix effects driven by chemical reactivity and electrostatic properties of the hosting substrate. The immobilization of two types of whole-cell luminescent bacterial sensors—a luminescent Cd-inducible reporter system and a constitutively luminescence-expressing strain—made it possible to investigate the aforementioned effects of hydrogel matrix on metal bioavailability and cell photoactivity performance while overcoming the limitations of conventional analytical techniques. The interpretation of the bioluminescence data measured for biosensors embedded in hydrogels or, for the purpose of comparison, dispersed in solution is based on a formalism built on a recent mechanistic theory describing how biosensor response integrates the cascade of extracellular and intracellular biophysicochemical processes leading to light production. Accordingly, a step-by-step data analysis was conducted using this theoretical framework so as to determine the extent to which hydrogel electrostatics, metal speciation and nutrient depletion impact the biosensor response within the hydrogel. A comparison between the response of luminescent bacterial sensors in solution and that of biosensors embedded in the hydrogels was then proposed by collating the maximum light emission per cell as a function of the total Cd-to-cell concentration ratio. In this interpretation scheme, metal partitioning at the hydrogel/solution interface was included and the electrostatic-driven accumulation of metals in the silica hydrogels was accounted for. In turn, a single master curve could be successfully generated for all bioluminescence data collected for biosensors embedded in hydrogels of varying thickness and different total biosensor concentrations. The analysis led to the evaluation of an electrostatic Boltzmann enrichment factor of Cd species in the hydrogels, which is in excellent agreement with that estimated independently from electrochemical speciation measurements. To further interconnect properly bioluminescence data measured within the hydrogel and solution scenarios, a second scaling procedure was applied to data pertaining to both situations in order to account for their differentiated metal speciation features, i.e., for the operational concentrations of bound and free Cd species (the latter referring to the supposedly internalizable metal form). Then, we evidenced that the representation of maximum light emission per cell *versus* free Cd-to-cell concentration ratio took the form of a remarkable single master curve where all bioluminescence data, whether measured in the solution or the hydrogel case, merged together. Under the examined conditions here, this important finding demonstrates that light production by biosensors mainly depends on free metal biouptake and by the electrostatic accumulation process. Despite a reduction in cell photoactivity in the hydrogels caused by a lack of nutrients renewal—a feature we evidenced by the analysis of the response of constitutively luminescent reporter systems—the loss of cell metabolic activity in the hydrogels appears to be of less importance compared to the contributions of metal speciation and the electrostatic accumulation process to the overall response of hydrogel-embedded biosensors.

**Figure 10:**
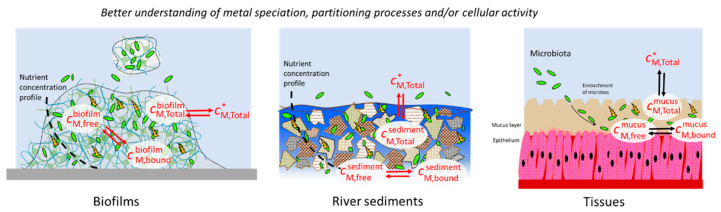
Illustrative examples of some possible applications of the concepts detailed in this work for the understanding of metal speciation and partitioning in heterogeneous environmental and biological matrices (biofilms, river sediments and tissues).

Following the successive data normalization procedures presented in this work, the distribution and bioavailability of target analytes can be here quantitatively assessed from analysis of only the maximum bioluminescence amplitudes, while the dependence of the biosensor response on time informs the variations of cell photoactivity. We evidence the key interplay between physicochemical metal speciation and electrostatic accumulation factors in governing the output of biosensors in hydrogels. Both factors depend on the chemical properties of the hosting matrix (e.g., presence of reactive charges and complexing sites) and on the metabolic properties of the biosensors when trapped in a confined environment. The analysis opens up new possibilities for investigating the biosensor response in heterogeneous biological and environmental microenvironments like tissues, sediments or biofilms (Figure 10), and refining our interpretation of biosensor signals to monitor toxicants/contaminants like drugs, pathogens or pesticides in such complex matrix systems. Furthermore, this work may contribute to the future design of original strategies aimed at understanding biotic and abiotic drivers of biofilm formation and functioning via the analysis of the response therein of dedicated inducible and/or constitutive biosensors. Moreover, the application of the biophysicochemical principles evidenced in this work from the reported controlled experiments will be possible providing that the analysis integrates, for example, (i) the heterogeneity of the biofilm composition that evolves over space and time, (ii) possible quorum sensing processes, and/or (iii) the transient partitioning — at the biofilm/solution interface — of the analytes that trigger the response of the selected inducible bioreporters. If items (i)–(iii) are captured properly, whole-cell reporter bacteria will then help in measuring, for example, cell metabolism and viability in growing biofilms, which are required for the monitoring of biofilms that lead to nosocomial infections or for the analysis of factors that affect the formation of environmental biofilms.

## Data Availability

All raw bioluminescence data reported in this work are available upon request.

## Supplementary Materials

The following supporting information can be downloaded at: https://www.mdpi.com/article/10.3390/bios14110552/s1. A: Reproducibility of experimental results: illustration for the time-dependent bioluminescence of gel-embedded biosensors measured at two different total Cd concentrations. B: Preparation of the thin mercury film electrode. C: Determination of the bound and free Cd concentrations in the nGGM 1:5 medium using the AGNES technique. D: Determination of the diffusion coefficient in the hydrogel using Crank’s model. E: Linear-Logarithmic representations of data given in Figure 7, Figure 8 and Figure 9. F: Analysis of the cell photoactivity contribution to the bioluminescence response of biosensors in the silica hydrogels of varied thicknesses. Figure S1: Example of experimental replicates illustrating the time-dependence of bioluminescence (*Lum*) produced by Cd-inducible whole-cell bioreporters JW3596-L in silica hydrogels at two selected values of total cadmium concentration, $c_{Cd,T}^{O}$= 20 nM and 40 nM (specified) for a gel thickness of 1143 µm; Figure S2: Time-dependence of cadmium concentration in the bulk solution (filled circles) and in the hydrogel phase (empty circles) for the three ionic strengths 10 mM (purple), 30 mM (orange) and 100 mM (green) for an initial Cd concentration in the bulk solution of 75 nM. The solution is nGGM 1:5 and ionic strength is adjusted with addition of NaNO_3_. The lines correspond to simulated curves using diffusion coefficients of (4.2 ± 2.0) × 10^−10^ m^2^ s^−1^, (6.20 ± 2.0) × 10^−10^ m^2^ s^−1^ and (3.72 ± 2.0) × 10^−10^ m^2^ s^−1^, λ = 0.53, 0.56, 0.75 ($V^{sol}\;$= 20 mL,$\;V^{gel}$ = 5.2 mL), *h* = 6 mm, for 10, 30 and 100 mM ionic strengths, respectively; Figure S3: Values of ${\mathrm{Lum}}_{max}/n_{\mathrm{cell}}$ as a function of ${r=c}_{Cd,T}^{O}/n_{\mathrm{cell}}$ for JW3596-L cells embedded in hydrogels of different thickness *h* (indicated, colored dots) or dispersed in solution (suspension case, black diamonds, indicated). The data were measured in nGGM 1:5 at pH 7.2. This figure displays the data of Figure 7, *albeit* according to a linear-logarithmic representation; Figure S4: ${\mathrm{Lum}}_{max}/n_{\mathrm{cell}}$ as a function of ${r\prime =c}_{Cd,T}^{x=gel,sol}/n_{\mathrm{cell}}$ for JW3596-L biosensors cells in hydrogels with varied thickness (colored dots, specified) or in solution (black diamonds, indicated) with the account of electrostatic Cd acumulation using $f_{B}=5$. The plot collects all the data obtained for the hydrogel and solution measurement configurations. Data were measured in nGGM 1:5 at pH 7.2. This figure displays the data of Figure 8, *albeit* according to a linear-logarithmic representation; Figure S5: As in Figure S4, except that ${\mathrm{Lum}}_{max}/n_{\mathrm{cell}}$ data are reported here as a function of *r*″ = $c_{\mathrm{Cd},\mathrm{free}}/n_{\mathrm{cell}}$. The grey shaded areas including all data points is a guide to the eye. This figure displays the data of Figure 9, *albeit* according to a linear-logarithmic representation; Figure S6: Ratio between ${\mathrm{Lum}}_{max}$ and number of biosensor cells in the sample (JW3596-L strain), $n_{\mathrm{cell}}$, as a function of $n_{\mathrm{cell}}$ for different values of the total Cd concentration $c_{Cd,T}^{O}$ (specified in the figure). The black dotted line represents the exponential variation of ${\mathrm{Lum}}_{c,\max}$ vs. $n_{\mathrm{cell}}$ evaluated for constitutive biosensors (JW3596-C) and displayed in Figure 4B of the main text. These data pertaining to constitutive cells are here converted into data plotted as a function of $n_{\mathrm{cell}}$ for the sake of comparison. The colored dotted lines represent the linear decrease of ${\mathrm{Lum}}_{\max}$ at the lowest $n_{\mathrm{cell}}$ values and highest Cd concentrations for Cd-inducible biosensor. References [57,58,59,60,61,62,63,64] are cited in the Supplementary Materials.

## Abbreviations

Latin Symbols	
a	Cell radius
Bn	Bosma number (dimensionless)
$c_{B,T}$	Total number concentration of bacteria applicable to the whole sample volume
$c_{B,T}^{gel}$	Total number concentration of bacteria in the silica hydrogels
$c_{B,p}^{gel}$	Number concentration of photoactive biosensors in the silica hydrogels
$c_{B,p}$	Number concentration of photoactive biosensors when dispersed in solution
${\bar{c}}_{B,p}$	$\mathrm{Dimensionless}\;\mathrm{concentration}\;\mathrm{of}\;\mathrm{photoactive}\;\mathrm{cells}\;\mathrm{defined}\;\mathrm{by}\;\mathrm{the}\;\mathrm{ratio}\;\mathrm{between}\;c_{B,p}\;$and the maximum number concentration of photoactive cells
$c_{B,p}^{\max}$	Maximum number concentration of photoactive cells
$c_{Cd,T}^{O}$	Total molar concentration of Cd in the whole sample volume
$c_{\mathrm{Cd},T}^{*}$	Total molar concentration of Cd in the solution positioned on top of the hydrogels (at equilibrium)
$c_{\mathrm{Cd},T}^{\mathrm{gel}}$	Total molar concentration of Cd concentration in the hydrogels
$c_{\mathrm{Cd},\;\mathrm{bound}}^{x}$	Molar concentration of bound Cd species in solution (x = solution) or in the hydrogels (x = gel)
$c_{\mathrm{Cd},\;\mathrm{free}}^{x}$	Molar concentration of free Cd ions in solution (x = solution) or in the hydrogels (x = gel)
$D_{Cd}$	Diffusion coefficient of Cd(II) in solution
$f_{B}$	Effective Boltzmann enrichment factor of Cd species in the hydrogels (at equilibrium)
h	Thickness of the hydrogel
$J_{u}$	Metal biouptake flux
${\left(K^{\prime }\right)}^{sol}$	Dimensionless thermodynamic stability constant of Cd complexes in solution
${\left(K^{\prime }\right)}^{\mathrm{gel}}$	Dimensionless thermodynamic stability constant of Cd complexes in the hydrogels
$K_{Hi}$	Hill constant, related to the affinity of the promoter (that controls the *lux*-reporter gene transcription) for the complex formed between intracellular Cd and the regulatory protein ZntR
$k_{int}$	Kinetic constant pertaining to the internalization of free metals via dedicated membrane transporter
$k_{\nu }$	Kinetic constant for photon emission per mole of luciferase
Lum	Bioluminescence produced by metal-detecting whole-cell bacterial sensors
${\mathrm{Lum}}_{c}$	Bioluminescence produced by constitutive whole-cell bacterial sensors
${\mathrm{Lum}}_{c,\mathrm{ini}}$	Basal level of bioluminescence produced by constitutive *lux*-biosensors
${\mathrm{Lum}}_{c,max}$	Maximum bioluminescence reached in the second emission regime for the constitutive *lux*-biosensors
${\mathrm{Lum}}_{max}$	Maximum bioluminescence reached in the second emission regime for the Cd-inducible biosensors
$n_{\mathrm{cell}}$	Total number of cells introduced in the sample
r	$\mathrm{Ratio}\;\mathrm{between}\;\mathrm{total}\;\mathrm{Cd}\;\mathrm{concentration},\;c_{Cd,T}^{o}$$,\;\mathrm{and}\;\mathrm{total}\;\mathrm{number}\;\mathrm{of}\;\mathrm{cells}\;(n_{\mathrm{cell}}$)
$r^{\prime }$	$\mathrm{For}\;\mathrm{the}\;\mathrm{hydrogel}\;\mathrm{case}:\;\mathrm{ratio}\;\mathrm{between}\;\mathrm{total}\;\mathrm{Cd}\;\mathrm{concentration}\;\mathrm{in}\;\mathrm{the}\;\mathrm{hydrogel},\;c_{Cd,T}^{gel}$$,\;\mathrm{and}\;\mathrm{total}\;\mathrm{number}\;\mathrm{of}\;\mathrm{cells},\;n_{cell}$; for the solution case: the ratio remains unchanged, i.e., r = $r^{\prime }$
$r^{\prime \prime }$	$\mathrm{Ratio}\;\mathrm{between}\;\mathrm{free}\;\mathrm{metal}\;\mathrm{concentration},\;c_{\mathrm{Cd},\mathrm{free}}$,$\;\mathrm{and}\;\mathrm{total}\;\mathrm{number}\;\mathrm{of}\;\mathrm{cells},\;n_{\mathrm{cell}}$
$S_{a}$	Surface area of an individual sensing cell
$S_{gel}$	Cross-sectional area of the hydrogel
$V^{gel}$	Volume of the hydrogel
$V^{sol}$	Volume of the solution on top of the hydrogel
$V^{T}$	Total sample volume
Greek Symbols	
γ	Henry coefficient for passive adsorption of Cd at the surface of the biosensors in solution
$\gamma_{gel}$	Henry coefficient for passive adsorption of Cd at the surface of the biosensors in the hydrogels
$\tau_{q}$	Characteristic timescale over which bioluminescence is emitted by a given luciferase–luciferin complex
φ	Gel volume fraction
