# Supplementary material for: Physicochemical Rationale of Matrix Effects Involved in the Response of Hydrogel-Embedded Luminescent Metal Biosensors
Source: Biosensors (Basel). 2024 Nov 13;14(11):552. doi: 10.3390/bios14110552 (PMC11591670; doi:10.3390/bios14110552)
Supplement: Supplementary file 1 [file biosensors-14-00552-s001.zip › biosensors-3262894-supplementary.pdf]

Supporting Material

Physicochemical rationale of matrix effects involved in the response of hydrogel-embedded luminescent metal biosensors

Elise Rotureau [1,*], Christophe Pagnout [2] and Jérôme F.L. Duval [1]

[1] Université de Lorraine, CNRS, LIEC, F-54000 Nancy, France ; jerome.duval@univ-lorraine.fr (J.F.L.D.)
[2] Université de Lorraine, CNRS, LIEC, F-57000 Metz, France ; christophe.pagnout@univ-lorraine.fr (C.P.)
* Correspondence: elise.rotureau@univ-lorraine.fr ; Tel.: +33-372-744-735 (E.R.)

This document contains 9 pages, 6 Supplementary Figures and 11 references.

A. Reproducibility of experimental results: illustration for the time-dependent bioluminescence of gel-embedded biosensors measured at two different total Cd concentrations

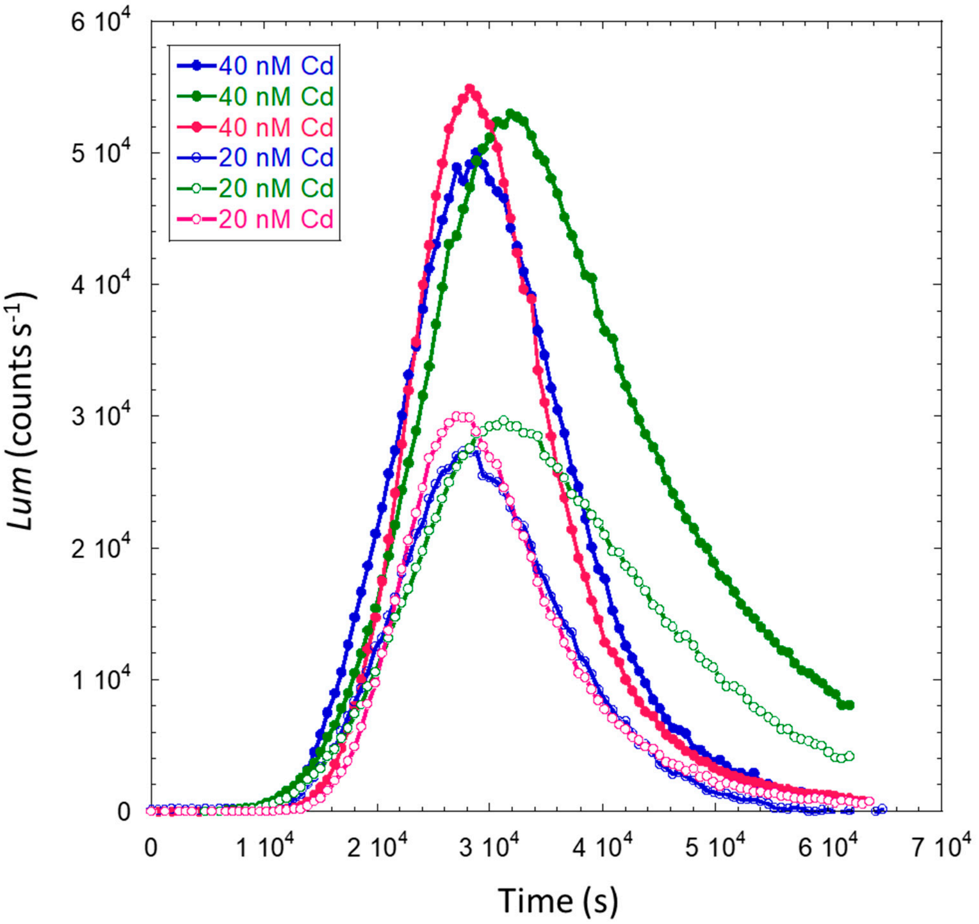

Figure S1. Example of experimental replicates illustrating the time-dependence of bioluminescence (*Lum*) produced by Cd-inducible whole-cell bioreporters JW3596-L in silica hydrogels at two selected values of total cadmium concentration, $c^{0}_{\mathrm{Cd,T}}$= 20 nM and 40 nM (specified) for a gel thickness of 1143 μm.

B: Preparation of the thin mercury film electrode

The protocol detailed here is based on the work published by Monterroso et al. [1] on rotating disk working electrode (glassy carbon disk). The first step consists in polishing the electrode surface using alumina (Metrohm) slurry for 1 min, followed by a thorough washing with ultrapure water. The second step consists in the electrochemical pre-treatment of the electrode via a series of 50 successive cyclic voltammograms between -0.800V and +0.800V *versus* Ag/AgCl at 0.1 V s^{-1} scan rate in NH_4Ac 1 M /HCl 0.5 M solution. The third step is the electrodeposition of the thin Hg film. For that purpose, the glassy carbon was immersed in a Hg(II) solution 0.24 mM (pH 1.9) and deposited by application of a potential of -1.300 V for 420 s with a rotation speed of 1000 rpm. After each working day, the charge associated with the deposited Hg was determined to assess the state of the mercury film. This was carried out by integration of the linear sweep stripping peak of Hg measured with a scan rate v = 0.005 V s^{-1} in 5 mM ammonium thiocyanide (pH 3.4) using a stripping potential range from -0.150 V to +0.400 V vs Ag/AgCl.

C: Determination of the bound and free Cd concentrations in the nGGM 1:5 medium using the AGNES technique

Absence of Gradients and Nernstian Equilibrium Stripping (AGNES) is a relevant electroanalytical technique designed for the determination of the free concentration of heavy metals. For more details about this technique, the reader is referred to the papers [2–4] explaining the AGNES measurement principles and detailing some application cases.

The nGGM 1:5 is mainly composed of various salts together with a buffering agent (MOPS) and glucose. The Cd equilibrium speciation in the presence of these chemicals are known and can be computed easily with available speciation codes such as Visual MINTEQ [5]. However, the only Cd complex formation constant with the β-glycerophosphate molecule present in nGGM is missing. Accordingly, we performed the following AGNES measurement to obtain the free and bound Cd amount in the nGGM diluted 5 times. A nGGM solution was initially prepared in the absence of β-glycerophosphate. This solution, diluted five times, was used to perform the AGNES calibration *i.e.* to establish the relationship between the AGNES signal and the free metal concentration. Then, the proper amount of glycerophosphate was added to reach a final concentration of 1 mM, and the quantification of the free Cd concentration was subsequently performed.

The same electrochemical equipment described in the main text is used here. Briefly, the three-electrode set-up is composed of a Dri-ref-5 electrode from WPI (Sarasota, FL, U.S.A.), a glassy carbon electrode and the working electrode is a thin mercury film plated onto a rotating glassy carbon disk of 2 mm diameter (Metrohm).

AGNES measurements were performed according to the following protocol. Metal deposition step at the mercury electrode was achieved by applying a potential E_d maintained constant during a suitable deposition time (t_d) under agitation conditions (1000 rpm rotation speed). The magnitude of the potential E_d was chosen in order to accumulate enough cadmium to be detected, and t_d was fixed so as to reach the situation in line with Nernstian equilibrium and absence of metal concentration gradients in the vicinity of the electrode surface [2,6,7]. Under our experimental conditions, E_d was fixed at -0.650V *vs.* Ag/AgCl and t_d at 100s. Regarding the stripping step, the oxidizing current was set to 3μA. At equilibrium, the gain (or preconcentration factor) Y is the ratio between the concentration of cadmium amalgamated in the mercury electrode and the concentration of the oxidized metal form in solution. It has been shown that the corresponding measured accumulated charge Q_{Cd} is proportional to the free metal concentration, which reads for cadmium as

$$Q_{\mathrm{Cd}} = Y\,\eta_Q\,c_{\mathrm{Cd,free}}^{\mathrm{sol}} \qquad \text{(S1)}$$

where $c_{\mathrm{Cd,free}}^{*}$ is the free Cd concentration in bulk solution, and η_Q is the proportionality factor defined by

$$\eta_Q = 2\,F\,V_{\mathrm{Hg}} \qquad \text{(S2)}$$

where F is the Faraday constant and V_{Hg} is the volume of the mercury electrode.

Calibration measurements consisted in preparing a 20 mL solution with the following composition: 4 mL of nGGM without β-glycerophosphate at pH 7.2 and 16 mL of ultrapure water. A disposable polystyrene cell was placed in a

double-walled container connected to a refrigerating-heating circulator and the temperature of the tested solution was set to 20°C. The solution was initially purged with nitrogen for 30 min, and a nitrogen blanket was systematically maintained afterwards above the sample solution. Firstly, a calibration plot was performed using the AGNES parameters (E_d; t_d) mentioned previously, with Cd concentrations ranging from 50 nM to 125 nM. By using the speciation computing code Visual MINTEQ [5], it was estimated that 88% of cadmium was in its free (unbound) form.

After calibration and addition of *β*-glycerophosphate solution, at least 4 AGNES measurements were performed and a mean value of Q_{Cd} was evaluated. The latter was used to evaluate the free Cd concentration. After addition of 1 mM glycerophosphate, the free Cd concentration decreased down to 70±1%, corresponding to an apparent dimensionless stability constant $(K')^{\mathrm{sol}} = c^{\mathrm{sol}}_{\mathrm{Cd,bound}}/c^{\mathrm{sol}}_{\mathrm{Cd,free}}$ of 0.42±0.02 for nGGM 1:5 medium.

D: Determination of the diffusion coefficient in the gel using Crank's model

For bioluminescence experiments, the hydrogel disks were disposed at the bottom of the wells. After solidification, the metal solution was added on top of the hydrogel and the microplate was gently and manually shaken for a few seconds. We then reproduced this setup at a larger spatial scale for the determination of the metal partitioning kinetics between the hydrogel and the solution, which led in turn to the determination of the diffusion coefficient of Cd ions in the silica hydrogel. The use of a hydrogel layer of several mm in thickness was required to minimize experimental errors on the quantification of the metal distribution between the hydrogel and the bulk of the external solution. The direct evaluation of metal depletion kinetics operating in the bulk solution once in contact with the hydrogel was achieved by means of the electroanalytical technique called Stripping Chronopotentiometry (SCP), as mentioned in the main text. Three different experiments were made at different ionic strengths by addition of $NaNO_3$ (1 M) in the 5-fold diluted nGGM medium, which defined the following salinity range: 10 mM for the nGGM (1:5) without added salt, 30 mM and 100 mM for nGGM supplemented with $NaNO_3$. The relationship between metal concentration in the bulk solution $c^*_{\mathrm{Cd,T}}$ at any time t with the one in the silica hydrogel over the whole volume, $c^{\mathrm{gel}}_{\mathrm{Cd,T}}$, from the mass balance condition.

Diffusion coefficients were then obtained from the time-dependence of $c^*_{\mathrm{Cd,T}}$ following the approach by Brown and Chitumbo [8] using the Crank equation under the form [9]:

$$\frac{c^*_{\mathrm{Cd,T}}(t)-c^*_{\mathrm{Cd,T}}(t\to\infty)}{c^*_{\mathrm{Cd,T}}(t=0)-c^*_{\mathrm{Cd,T}}(t\to\infty)} = \sum_{n=1}^{\infty} \frac{2\lambda(1+\lambda)}{1+\lambda+\lambda^2 q_n^2} \exp\left(-\frac{4q_n^2 D_{\mathrm{Cd}} t}{h^2}\right) \qquad \text{(S3)}$$

with $c^*_{\mathrm{Cd,T}}(t=0)$ the initial metal concentration in the solution, *i.e.* the total metal concentration in the bulk solution at t=0, $c^*_{\mathrm{Cd,T}}(t)$ and $c^*_{\mathrm{Cd,T}}(t\to\infty)$ the concentration of Cd at time t and at $t\to\infty$, h the layer thickness of the disk-shaped silica gel. The q_n^2 values are successive, non-zero, positive roots of the equation

$$\tan q_n = -\lambda\, q_n \qquad \text{(S4)}$$

and λ is the effective volume ratio given by:

$$\lambda = \frac{V^{\mathrm{sol}}}{f_{\mathrm{B}} V^{\mathrm{gel}}} \qquad \text{(S5)}$$

where f_{B} is the electrostatic partition coefficient (see details in the main text) found to be 10.3±0.5, 8.9±0.5, 5.6±0.5 for 10 mM, 30 mM and 100 mM ionic strength, respectively. As expected, values of f_{B} decrease with increasing medium salinity as a result of the screening of hydrogel charges by ions [10].

Variations of the metal concentration with time are exponential-like, so that equation 3 above can be reduced to one single exponential term (n=1).

Plotting the linear variation of $\log \frac{c^*_{\mathrm{Cd,T}}(t)-c^*_{\mathrm{Cd,T}}(t\to\infty)}{c^*_{\mathrm{Cd,T}}(t=0)-c^*_{\mathrm{Cd,T}}(t\to\infty)} = f(t)$ with time, the intercept provides the q_1 values for a given λ. Then, the diffusion coefficient is obtained from the value of the slope. For a hydrogel layer thickness of 6 mm, we found a mean diffusion coefficient of $(4.2\pm2.0)\times10^{-10}$ m^2 s^{-1}, $(6.20\pm2.0)\times10^{-10}$ m^2 s^{-1} and $(3.72\pm2.0)\times10^{-10}$ m^2 s^{-1} for 10 mM, 30 mM and 100 mM solution ionic strength, respectively. The corresponding experimental and

computed time-dependent metal concentrations in bulk solution, $c^*_{\mathrm{Cd,T}}$, and over the whole gel volume, $c^{\mathrm{gel}}_{\mathrm{Cd,T}}$, are displayed in **Figure S2**. We found that the evolution of $c^*_{\mathrm{Cd,T}}$ with time is very similar under the three tested ionic strength conditions, which suggests that the attractive electrostatics between the negatively charged hydrogel and the metal cations has poor accelerating effect on the diffusion of the metal species. This finding agrees with the theoretical prediction by Duval et al. [11] under the condition where the dimension of the accumulating phase (here the hydrogel) is much larger than the thickness of the electric double layer. This condition is obviously verified in our study as hydrogels are mm-thick and the thickness of electric double layers does not exceed here 3 nm.

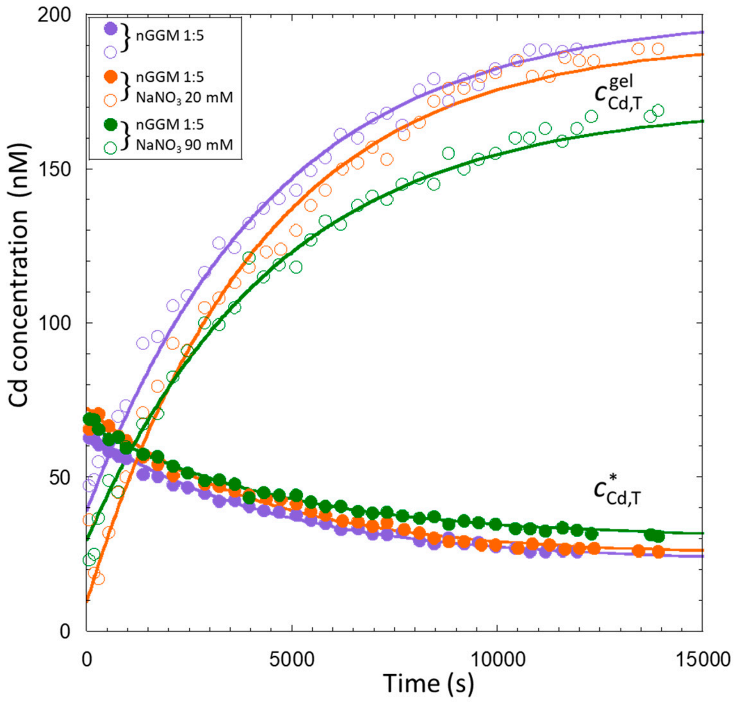

Figure S2. Time-dependence of cadmium concentration in the bulk solution (filled circles) and in the hydrogel phase (empty circles) for the three ionic strengths 10 mM (purple), 30 mM (orange) and 100 mM (green) for an initial Cd concentration in the bulk solution of 75 nM. The solution is nGGM 1:5 and ionic strength is adjusted with addition of $NaNO_3$. The lines correspond to simulated curves using diffusion coefficients of (4.2±2.0)×10^{-10} m^2 s^{-1}, (6.20±2.0) ×10^{-10} m^2 s^{-1} and (3.72±2.0)×10^{-10} m^2 s^{-1}, λ=0.53, 0.56, 0.75 (V^{sol} = 20 mL, V^{gel} = 5.2 mL), h = 6 mm, for 10, 30 and 100 mM ionic strengths, respectively.

The effective diffusion coefficient of Cd in the silica hydrogel is of the same order of magnitude than the one in water (6.37×10^{-10} m^2 s^{-1} at 25°C) and the one in the polyacrylamide gel (4.02×10^{-10} m^2 s^{-1} at 25°C, in 10 mM ionic strength).

E: Linear-Logarithmic representations of data given in Figures 7, 8 and 9

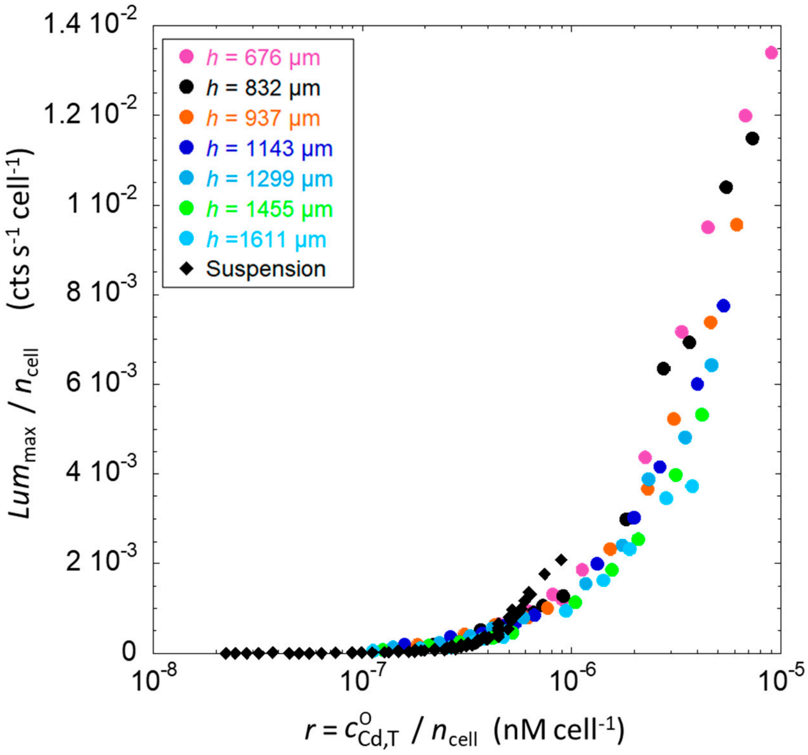

Figure S3. Values of $Lum_{\mathrm{max}}/n_{\mathrm{cell}}$ as a function of $r = c^{\mathrm{O}}_{\mathrm{Cd,T}}/n_{\mathrm{cell}}$ for JW3596-L cells embedded in hydrogels of different thickness h (indicated, colored dots) or dispersed in solution (suspension case, black diamonds, indicated). The data were measured in nGGM 1:5 at pH 7.2. This figure displays the data of **Figure 7**, *albeit* according to a linear-logarithmic representation.

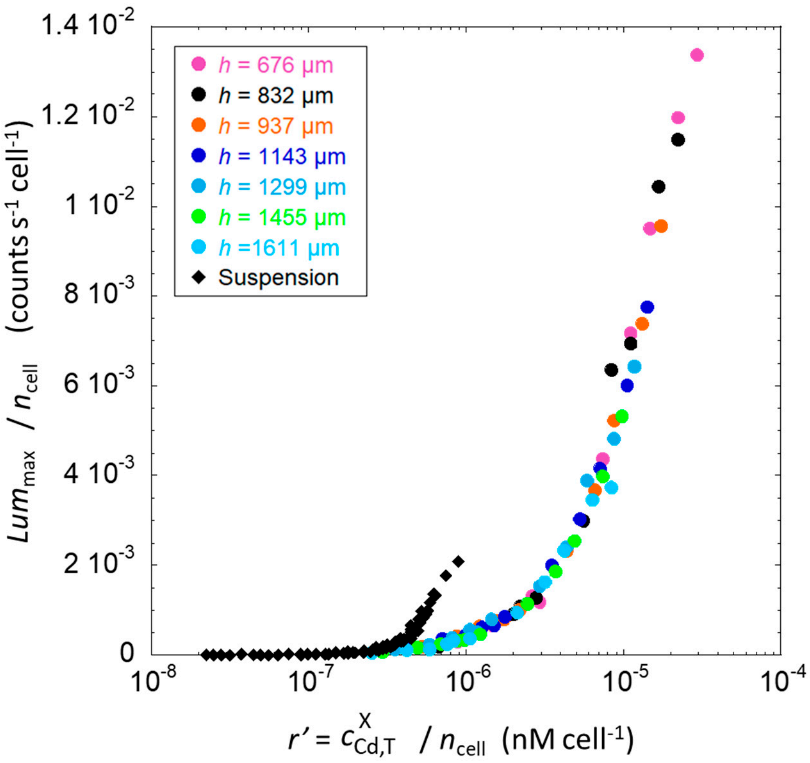

Figure S4. $Lum_{\mathrm{max}}/n_{\mathrm{cell}}$ as a function of $r' = c_{\mathrm{Cd,T}}^{\mathrm{x=gel,sol}}/n_{\mathrm{cell}}$ for JW3596-L biosensors cells in hydrogels with varied thickness (colored dots, specified) or in solution (black diamonds, indicated) with the account of electrostatic Cd acumulation using $f_{\mathrm{B}} = 5$. The plot collects all the data obtained for the hydrogel and solution measurement configurations. Data were measured in nGGM 1:5 at pH 7.2. This figure displays the data of **Figure 8**, *albeit* according to a linear-logarithmic representation.

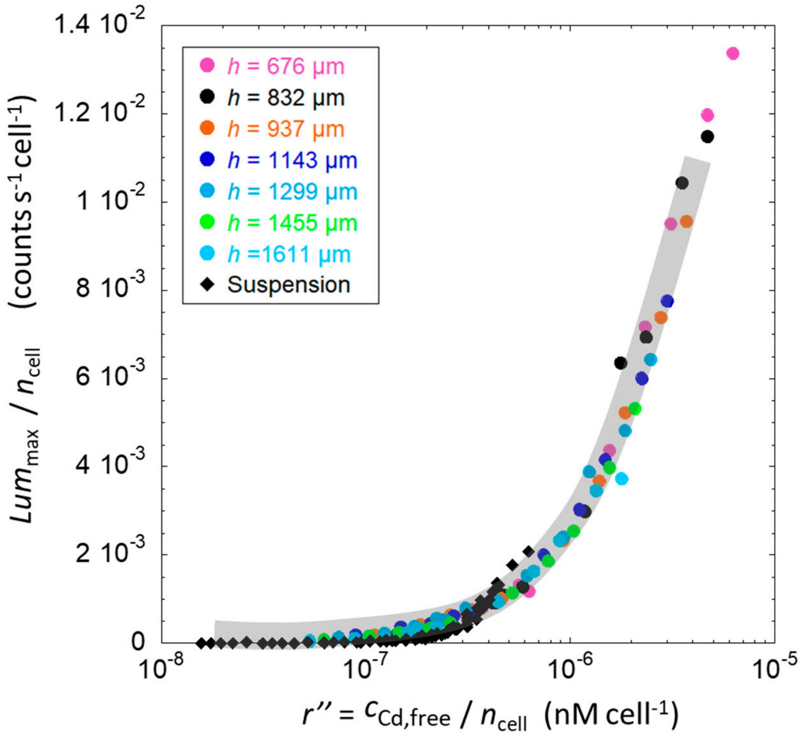

Figure S5. As in Figure S4, except that Lum_{max}/n_{cell} data are reported here as a function of $r''=c_{Cd,free}/n_{cell}$. The grey shaded areas including all data points is a guide to the eye. This figure displays the data of **Figure 9**, *albeit* according to a linear-logarithmic representation.

F: Analysis of the cell photoactivity contribution to the bioluminescence response of biosensors in the silica hydrogels of varied thicknesses

We analyze here the relative variations of Lum_{max} and $Lum_{c,max}$ per cell as a function of n_{cell} for the two strains of interest in our work, *i.e.* the luminescent Cd-inducible reporter system and the constitutively luminescent strain. In **Figure S6**, Lum_{max}/n_{cell} is then reported for each total Cd concentration, $c^{O}_{Cd,T}$, while the only exponential-like variation of $Lum_{c,max}/n_{cell}$ with n_{cell} is given (cf. dotted line in **Figure S6**), because Cd concentration has a negligible effect on the response of constitutive *lux*-biosensor (cf. details in the main text). The corresponding exponential law corresponds to that displayed in **Figure 5B** of the main text.

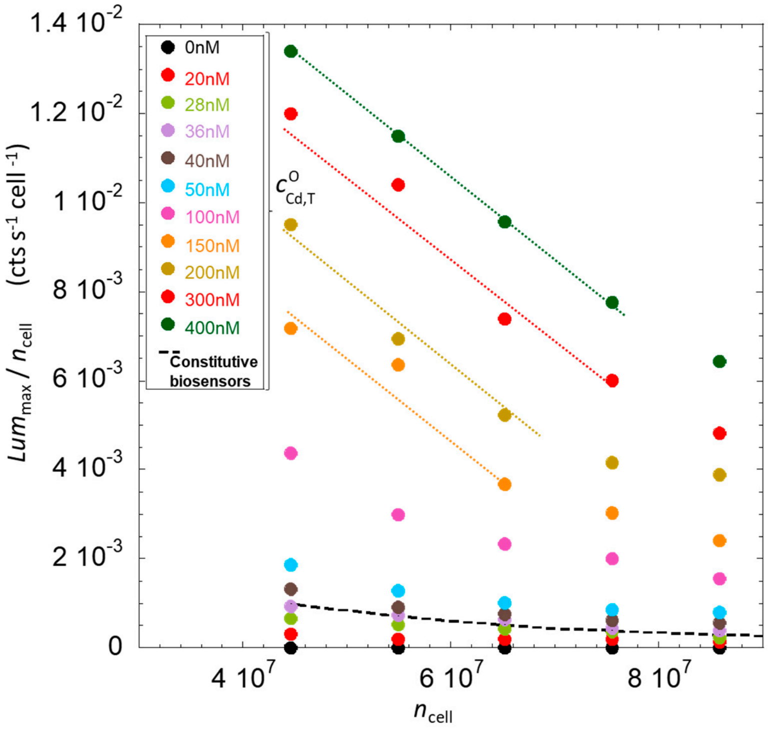

Figure S6. Ratio between Lum_{max} and number of biosensor cells in the sample (JW3596-L strain), n_{cell}, as a function of n_{cell} for different values of the total Cd concentration $c^{O}_{Cd,T}$ (specified in the figure). The black dotted line represents the exponential variation of $Lum_{c,max}$ *vs.* n_{cell} evaluated for constitutive biosensors (JW3596-C) and displayed in Figure 4B of the main text. These data pertaining to constitutive cells are here converted into data plotted as a function of n_{cell} for the sake of comparison. The colored dotted lines represent the linear decrease of Lum_{max} at the lowest n_{cell} values and highest Cd concentrations for Cd-inducible biosensor.

For $c^{O}_{Cd,T} \leq 50$ nM, the decrease of the bioluminescence per cell for Cd-inducible biosensors with increasing n_{cell} is comparable in magnitude with the one observed for the constitutive biosensors. In contrast, for $c^{O}_{Cd,T} \geq 100$ nM, this decrease is significantly larger than the one featured by the exponential decay reported for the JW3596-C (dotted line in **Figure S6**). This suggests that the loss in cell photoactivity is not the primary cause in the reduction of bioluminescence production by Cd-inducible biosensors. For the highest total Cd concentrations tested, we may differentiate two distinct regimes: a linear decrease that operates at low amounts of cell, n_{cell}, followed by a second regime at larger n_{cell} where linearity is lost and the decrease in Lum_{max}/n_{cell} becomes less marked. The linear regime is simply explained by the passive adsorption of metals at the surface of the biosensors, which corresponds to the term $1 - \gamma_{gel}\, S_a c^{gel}_{B,T}$ in Equation 8 of the main text, recalling that n_{cell} is directly proportional to $c^{gel}_{B,T}$. The linearity of the data sets in **Figure S6** at sufficiently low n_{cell} thus indicates that the change in bioluminescence per cell in that regime is caused by the only prefactor of Equation 8 in the main text and that the convolution product in that equation is likely constant over the corresponding range of n_{cell}. Therefore, the variations in cell photoactivity within the linear regime do not contribute predominantly to the reduction in Lum_{max}/n_{cell} in that regime. Outside the linear regime, at larger n_{cell}, it is reasonable to think that M passive sorption is so important that additional processes may significantly impact on the dependence of the bioluminescence produced per cell on n_{cell}. The loss

of linearity could then reflect that the convolution product is no longer constant within this range of n_{cell}. From the comparison of the results obtained for the constitutive and Cd-inducible biosensors, it can be concluded that the loss in cell photoactivity discussed in section 4.2.2 of the main text does not explain the variations of bioluminescence maxima measured with varying hydrogel thickness. Accordingly, there is no need to figure out additional corrections of the data in **Figure 9** so as to generate a master curve of better quality than the one already achieved following the procedure detailed in the main text.

References

1. Monterroso, S.C.C.; Carapuca, H.M.; Simao, J.E.J.; Duarte, A.C. Optimisation of Mercury Film Deposition on Glassy Carbon Electrodes: Evaluation of the Combined Effects of pH, Thiocyanate Ion and Deposition Potential. *Anal. Chim. Acta* **2004**, *503*, 203–212, doi:10.1016/j.aca.2003.10.034.
2. Galceran, J.; Companys, E.; Puy, J.; Cecilia, J.; Garces, J.L. AGNES: A New Electroanalytical Technique for Measuring Free Metal Ion Concentration. *J. Electroanal. Chem.* **2004**, *566*, 95–109, doi:10.1016/j.jelechem.2003.11.017.
3. Chito, D.; Weng, L.; Galceran, J.; Companys, E.; Puy, J.; van Riemsdijk, W.H.; van Leeuwen, H.P. Determination of Free Zn2+ Concentration in Synthetic and Natural Samples with AGNES (Absence of Gradients and Nernstian Equilibrium Stripping) and DMT (Donnan Membrane Technique). *Sci. Total Environ.* **2012**, *421–422*, 238–244, doi:10.1016/j.scitotenv.2012.01.052.
4. Domingos, R.F.; Huidobro, C.; Companys, E.; Galceran, J.; Puy, J.; Pinheiro, J.P. Comparison of AGNES (Absence of Gradients and Nernstian Equilibrium Stripping) and SSCP (Scanned Stripping Chronopotentiometry) for Trace Metal Speciation Analysis. *J. Electroanal. Chem.* **2008**, *617*, 141–148, doi:10.1016/j.jelechem.2008.02.002.
5. Gustafsson, J.P. Visual MINTEQ Version 3.0. KTH, Department of Land and Water Resources Engineering, Stockolm, Sweden, 2009. Available at Http://Vminteq.Lwr.Kth.Se/.
6. Rotureau, E.; Pla-Vilanova, P.; Galceran, J.; Companys, E.; Pinheiro, J.P. Towards Improving the Electroanalytical Speciation Analysis of Indium. *Anal. Chim. Acta* **2019**, *1052*, 57–64, doi:10.1016/j.aca.2018.11.061.
7. Tehrani, M.H.; Companys, E.; Dago, A.; Puy, J.; Galceran, J. Free Indium Concentration Determined with AGNES. *Sci. Total Environ.* **2018**, *612*, 269–275, doi:10.1016/j.scitotenv.2017.08.200.
8. Brown, W.; Chitumbo, K. Solute Diffusion in Hydrated Polymer Networks. Part 1.—Cellulose Gels. *J. Chem. Soc., Faraday Trans. 1* **1975**, *71*, 1, doi:10.1039/f19757100001.
9. Crank, J. *The Mathematics of Diffusion*; 2d ed.; Clarendon Press: Oxford, UK, 1975; ISBN 978-0-19-853344-3.
10. Kalis, E.J.J.; Davis, T.A.; Town, R.M.; Van Leeuwen, H.P. Impact of Ionic Strength on Cd(II) Partitioning between Alginate Gel and Aqueous Media. *Environ. Sci. Technol.* **2009**, *43*, 1091–1096, doi:10.1021/es802305n.
11. Duval, J.F.L.; van Leeuwen, H.P. Rates of Ionic Reactions With Charged Nanoparticles In Aqueous Media. *J. Phys. Chem. A* **2012**, *116*, 6443–6451.
